# Incentives for Research Effort: An Evolutionary Model of Publication Markets with Double-Blind and Open Review

**DOI:** 10.1007/s10614-022-10250-w

**Published:** 2022-04-08

**Authors:** Mantas Radzvilas, Francesco De Pretis, William Peden, Daniele Tortoli, Barbara Osimani

**Affiliations:** 1 Department of Philosophy, University of Konstanz, 78464 Konstanz, Germany; 2 Department of Communication and Economics, University of Modena and Reggio Emilia, 42121 Reggio Emilia, Italy; 3 Erasmus Institute for Philosophy and Economics, Erasmus University Rotterdam, 3062 PA Rotterdam, The Netherlands; 4 Department of Biomedical Sciences and Public Health, Marche Polytechnic University, 60126 Ancona, Italy

**Keywords:** Agent-based model, Double-blind peer review, Evolutionary game theory, Open review, Publication markets, Simulation, 91A22, C63, C73, D47, D58

## Abstract

Contemporary debates about scientific institutions and practice feature many proposed reforms. Most of these require increased efforts from scientists. But how do scientists’ incentives for effort interact? How can scientific institutions encourage scientists to invest effort in research? We explore these questions using a game-theoretic model of publication markets. We employ a base game between authors and reviewers, before assessing some of its tendencies by means of analysis and simulations. We compare how the effort expenditures of these groups interact in our model under a variety of settings, such as double-blind and open review systems. We make a number of findings, including that open review can increase the effort of authors in a range of circumstances and that these effects can manifest in a policy-relevant period of time. However, we find that open review’s impact on authors’ efforts is sensitive to the strength of several other influences.

## Introduction

One of science’s strengths is that its institutions and methods are revised in response to experience and analytical criticisms. Today, there are many proposals for reforming various institutions within scientific disciplines. These are partly stimulated by the discovery of the Replication Crisis, in which research in a number of fields—like social psychology and medical research—replicates at a far lower rate than expected (Ioannidis, 2005; Fidler & Wilcox, 2018; Open Science Collaboration, 2015).^1^ Proposals for change include modifications to statistical methodologies and concepts (Benjamin et al., 2017; Gelman, 2015; Mayo, 2018; Radzvilas et al., 2021; Trafimow, 2018; Wasserstein & Lazar, 2016) and greater transparency (AllTrials, 2014; Center for Open Science, 2013). Yet, even before the Replication Crisis revelations, there were discussions about scientific institutions and practices. For instance, there are fundamental debates about hierarchies of evidence (Cartwright, 2007; Worrall, 2002) and the proper role of statistical significance testing (Ziliak & McCloskey, 2008). A common theme in most of these debates is that, even when scientists are not being dishonest or illogical, relatively low-effort practices can lead to systematic problems and wasted resources. Furthermore, the many proposed methodological and institutional reforms tend to require increased effort (at least per-paper) by scientists. This effort might be achieved by ethical reasoning and other forms of persuasion, but we also have to consider how the professional incentives of scientists either encourage or discourage spending effort on a paper.

However, scientists’ incentives for expending effort in research are complex, and therefore the effects of institutional reforms on encouraging above-average effort per-paper are not easily predicted. It is thus worth pursuing systematic models of how different institutional environments provide incentive structures for scientists (Maniadis & Tufano, 2017). In this article, we focus on a topic that has lacked a specific and systematic formal economic study: how peer review shapes incentives for research effort. This issue has been described as perhaps the most pressing question in the social study of science (Franklin, 2009, p. 203). In particular, we are interested in the challenge of incentivising scientists who are already reasonably responsible and able to “pass the desk” at quality journals. We focus on encouraging exemplary effort over merely adequate effort. It is the former that science needs in the prospective future in order to respond to challenges like the Replication Crisis. Thus, we leave aside the issue of disincentivising outright junk science.

As every scientist knows, a large part of their incentives can be summarised as “publish or perish”. Publication records are crucial factors in hiring decisions, grant approvals, conference invitations, and their general reputation. However, it is normally impossible for decision-makers to read and systematically evaluate all of a scientist’s research. In particular, assessors will often lack the skills, time, and resources to evaluate the work of a scientist who has published across multiple disciplines or across a wide range of topics. Thus, they will often resort to evaluating the scientist’s research output on the basis of metrics that they regard as proxies for research quality, such as journal rankings, impact factors, and researcher citation metrics. These metrics can be exploited with quantity-over-quality strategies.

Fortunately, there are institutional pressures that might help limit these malincentives. One of these is peer review. If submitting relatively low-effort research almost always led to rejections, then a scientist who tried to produce a high quantity of such research would have almost no publications, whereas scientists who focused on producing fewer high-effort research would be comparatively advantaged in hiring decisions, funding approvals, and so on. Yet the incentive structures of peer review are still largely mysterious.

There are many different types of peer review systems, which vary in multiple dimensions. A major dimension of difference is the blinding procedure. For example:

1. Triple-blind: authors’ identities are hidden to both reviewers and editors; reviewers’ identities are hidden to authors.
2. Double-blind: the identities of both authors and reviewers are hidden to each other.
3. Single-blind: the identities of reviewers are hidden to authors, but not vice versa.
4. Open review: for accepted papers, no identities are hidden. All identities are hidden when papers are rejected. The identities of authors may or may not be hidden during review.^2^

In this article, we focus on the effects of peer review systems on reputation incentives and effort costs of authors and reviewers, rather than holistically evaluating peer review systems. There are already debates about how these systems can affect reviewer bias (Armstrong, 1997; Bernard, 2018; Budden et al., 2008; Cox et al., 1993; Darling, 2014; Engqvist and Frommen, 2008; Ferber and Teiman, 1980; Fish, 1989; Garvalov, 2015; Largent & Snodgrass, 2016; Lotfi & Mahian, 2014; Tomkins et al., 2017; Webb et al., 2008) and reviewer behaviour towards authors (Comer & Schwartz, 2014; Turner, 2003; Wendler & Miller, 2013). There is also a small informal literature on the effects of peer review systems on reviewers’ incentives for good practices (Chubin & Hackett, 1990; Eisenhart, 2002; Godlee, 2002; Heesen & Bright, 2021; Raelin, 2008; Rowbottom, 2021; Wendler & Miller, 2013). While this literature is valuable, it lacks the systematic rigour that formal tools such as game theory can provide. There has been some empirical study into the effects of peer review systems (Leek et al., 2011), but only with respect to cooperation between reviewers and authors. Blinded and non-blinded evaluation has been investigated, but not specifically for publication markets, and with a focus on the effects of subjective character information (Taylor & Yildirim, 2011).

In contrast to these earlier studies, our model covers both authors and reviewers, and uses the tools of game theory to explore the connections between competing incentives and interactive strategies. It thus makes a very novel contribution to the debates on peer review systems and provides the basis for further systematic modelling of their incentive effects on research effort.^3^

We found that existing agent-based models of peer review did not fully meet our requirements. For example, Wang et al. (2016) develop a model that includes the effects of reviewer choices on the quality of published papers. However, in their model, authors cannot choose their effort levels on a paper, nor can reviewers choose their thresholds for accepting papers. Thus, their model is unable to answer questions about the consequences of institutions on strategic behaviour, since there are no strategic choices by authors and reviewers in their model. Therefore, we have developed a model in which the strategies of authors and reviewers are not fixed: authors and reviewers can revise their peers strategies, observe their performance in previous interactions, and use this newly acquired information to adapt their behaviour in reaction to the institutional environment.

Similarly, Bianchi et al. (2018) study the effects on peer review quality of authors and reviewers making choices about their time allocations between (a) authoring activities and (b) reviewing activities. Unlike that study, we consider the subjectively assessed general effort costs of authors and reviewers. Additionally, we focus on choices regarding the effort costs of authoring and reviewing individually, rather than the trade-off between these two activities. Put differently, we consider their decisions regarding the trade-off between these particular roles and *all* their alternative activities, including leisure. Finally, as in the model of Wang et al. (2016), players in Bianchi et al. (2018) cannot switch between strategies. Therefore, their model cannot answer questions about the evolutionary competition of different strategies over time. Overall, despite the merits of both models, they were not suitable for our purposes.

In Sect.  2, we explain our base game, the agent-based model that we used, and the process behind our simulations. In Sect.  3, we discuss our results. Finally, in Sect.  4, we connect our simulations to broader questions and consider some of our study’s limitations, before concluding in Sect.  5.

## Methods

In this section, we describe our model and its underlying game. Our ultimate aim is a simple exploratory model of the incentives for high effort expenditures by authors. Towards this goal, we also model the effort expenditure decisions of reviewers. We focus on author effort rather than research because there is no consensus on how to quantify the latter or relate it to authors’ decisions, whereas it is reasonable to model the former as a single parameter that is directly under authors’ control.

We focus on high versus relatively average effort because the filtering of low-effort papers is mostly performed by editors, who do not feature in our model at this stage in its development. Our model can be interpreted as making the idealization that editors always “desk reject” low-effort papers.^4^ For the surviving papers, we assume that editors follow their reviewers’ recommendations, and hence we can just model the judgements of reviewers. This assumption’s acceptability is supported by an empirical study (Card & DellaVigna, 2020), which finds that economics editors follow referees’ recommendations closely. We also assume that journal submissions are reviewed by a single reviewer. This assumption is not realistic, but it does correspond to what authors principally care about, which is the aggregation of the reviewers’ judgements. Since our ultimate interest is the effort expended by authors, this assumption does not seem to hinder our main aims.

Additionally, we use boundedly but almost rational (i.e. personal payoff maximising) players. To represent the strategy choices of these players, we explore the model’s evolutionary dynamics under several strategy revision algorithms. The first is standard best response dynamics (Gilboa and Matsui, 1991) that models the interacting players as decision-makers who revise their strategies by always picking the considered strategy with the highest historical average payoff. In multi-population games with random matching, such as our own, quasistrict Nash equilibria^5^ are regular evolutionarily stable states under best response dynamics.^6^ These convergence properties of best response dynamics make it a useful benchmark case in our analysis for comparison with more realistic models that introduce stochastic elements into the players’ strategy revision process.

We also used logit response dynamics (Blume, 1993) to model a strategy revision process with *endogenous* noise – players’ occasional failures to choose strategies with the highest average historical payoffs. (This type of noise is distinct from random influences that are external to the players, such as funding problems, which we represent separately.) A logit choice rule assigns a probability to each considered strategy, and the probability is based on its relative performance in previous rounds of the game. The considered strategies with the highest historical average payoff are assigned the highest probability of being selected by the player. Yet the rule also assigns a smaller—but positive—probability to considered strategies with lower-than-maximum historical average payoffs. Thus, each strategy revising player has a nonzero probability of choosing every considered strategy. The analysis of the author-reviewer games that were generated by our choices of parameter values revealed that none of these games were potential or near-potential games.^7^ Consequently, it was not possible to apply the currently available analytical techniques that can be used to determine the stochastically stable population states under logit response dynamics in potential games.^8^

Additionally, as we explain below, our model yields author-reviewer games with multiple Nash equilibria in pure strategies. Therefore, simulations offer a useful tool for exploring the properties of the model by providing its evolutionary dynamics (the process by which the populations converge to an evolutionarily or stochastically stable state) which would not be derivable from an analytical study. Since logit response dynamics is a smooth approximation of best response dynamics, simulations of logit response dynamics and its comparison with best response dynamics provided an unusually appealing approach towards the analysis of the convergence of populations under logit response dynamics.^9^ Given these population dynamics with best response and logit choice models, we also introduced best response and logit response models with a small degree of *exogenous* noise – a small probability of a strategy-revising player temporarily abandoning their decision rule (whether best response or logit choice) and choosing the next strategy randomly. This stochastic feature may be interpreted as a means of incorporating unpredictable exogenous factors that might incentivise or disincentivise effort expenditures by authors, such as whether an author is approaching a tenure decision. However, authors are still myopic payoff maximisers, because we are interested in identifying the effects of parameter values and institutional settings (such as different review systems) on the players’ strategic choices. Similarly, for reviewers, we incorporate a small probability that reviewers will adopt a less historically successful threshold as their standard. This stochastic element in reviewers’ behaviour can be interpreted as capturing a variety of random exogenous factors that could affect reviewers’ methodological standards. It is another reason for our choice of simulation methods over a purely analytical study. See Sect.  2.2 for more discussion of this stochastic element in our model.

We explain our model as follows: (1) the base game that schematises a particular author-reviewer interaction and (2) our agent-based model that studies how the strategies in the author and reviewer populations evolve over time under different initial conditions, i.e. parameters and institutional settings. Finally, we detail our simulation process and its outputs.

### The Author-Reviewer Game

The base game is a two player $6\times 6$ normal form game^10^ which represents the one-shot interaction between a player *Au* denoting an author and a player *Re* embodying a reviewer. We assume that, in order to pass the editorial desk and enter a peer review process, the author’s effort level must be equal or higher than the minimum threshold ${\underline{t}}\in \left( 0,1\right)$. *Au*’s set of strategies is a finite set of effort levels $E=\left\{ e_{1},\ldots,e_{6}\right\}$, such that $e_{i}\ne e_{j}$ for every pair $e_{i}\in E$ and $e_{j}\in E$, and each $e_{i}:={\underline{t}}+x$, where $x\in \left[ 0,1-{\underline{t}}\right]$ is the amount of additional effort chosen by *Au*. Notice that the maximum possible effort which the author could choose is 1.

*Re*’s strategies is a set $R:=T\times \left\{ d\right\}$ with a typical element $r_{k}=\left( t_{m},d\right)$. We unpack *R*’s definition in two steps. Firstly, $T=\left\{ t_{1},...,t_{6}\right\}$ is a set of acceptance thresholds, such that $t_{m}\ne t_{n}$ for every pair $t_{m}\in T$ and $t_{n}\in T$, and where each $t_{m}:={\underline{t}}+y$, where $y\in \left[ 0,1-{\underline{t}}\right]$ is the additional effort demand chosen by *Re*. Secondly, $d:E\times T\rightarrow \left\{ a,{\tilde{a}}\right\}$ is *Re*’s response function which assigns, to every effort-threshold pair $\left( e_{i},t_{m}\right) \in E\times T$, a response *a* (recommendation to accept) if $e_{i}\ge t_{m}$ or ${\tilde{a}}$ (recommendation to reject) if $e_{i}<t_{m}$. Notice we assume that the maximum possible acceptance threshold is 1. Thus, the reviewer recommends the editor to accept the author’s paper if it meets or exceeds the reviewer’s requirements for effort, and recommends the editor to reject the paper if it does not. The editor always follows the reviewer’s recommendation.

We model *Au*’s incentives in such a way that there are both costs and benefits to expending effort on their research. *Au*’s base payoff from acceptance of the paper is 1, while rejection yields a base payoff equal to 0. The acceptance base payoff represents the pure reputation bonus from publishing in a good journal, while the rejection base payoff represents the absence of this bonus. In addition, *Au* receives payoff bonuses which depend on *Au*’s effort level and *Re*’s acceptance threshold. The author’s effort level is important for their career, because it is a proxy for article quality, which tends to positively impact an author’s reputation, and because it is costly. The reviewer’s acceptance threshold determines, for a given author effort level, whether the article is accepted, as any author effort level that exceeds the reviewer’s threshold will be published, and otherwise the author’s paper is rejected.

*Au*’s effort is costly and the cost is proportional to the invested effort: higher effort is associated with a higher effort cost than lower effort. Formally, *Au*’s effort cost will be defined as $\beta e_{i}$, where $\beta >0$. We define these costs in the ordinary way: they are the total opportunity costs of spending effort on the particular journal submission rather than alternative uses of *Au*’s time, funding, energy, and so on.^11^ Thus, $\beta$ includes all author cost constraints, which become binding when they exceed *Au*’s expected benefits from effort expenditures. They are also constraining in that the benefits (and costs) from publication are represented by bounded variables. Therefore, there is always a point where the costs of effort expenditure exceed the benefits; we do not assume that *Au* has an infinite capacity or willingness for effort.

In case of acceptance, *Au* gets a reputation boost represented by a payoff bonus $\epsilon e_{i}$, where $e_{i}\in E$ is *Au*’s chosen effort level and $\epsilon \ge 0$. It is thus the reputation benefits that *Au* obtains from their article. We assume this bonus to be proportional to *Au*’s effort level. If *Au*’s payoff from acceptance is sufficiently high, then the author has an incentive to invest above average effort independently of reviewer behaviour. Our interest is when reputation incentives for author effort are insufficient to guarantee high author effort – it is under such circumstances that peer review has an important role of incentivising effort. Given the effort and threshold values that we investigated, we found in simulations that the separation of reviewer behaviour and author effort occurred when $\epsilon \ge 0.2$. Therefore, we limit our inquiry in this article to $\epsilon \le 0.2$.

By combining all the aforementioned incentives, we can define *Au*’s final payoffs with a payoff function $\pi _{Au}:E\times R\rightarrow {\mathbb {R}}$, which is such that

1 $$\begin{aligned} \forall \left( e_{i},r_{k}=\left( t_{m},d\right) \right) \in E\times R, \pi _{Au}\left( e_{i},r_{k}\right) =\left\{ \begin{array}{ll} 1 +\epsilon e_{i}-\beta e_{i} &\quad \text{ when } e_{i}\ge t_{m}; \\ 0-\beta e_{i}& \quad \text{ otherwise. } \end{array}\right. \end{aligned}$$

Thus, *Au* receives positive utility from acceptance, quantified by the positive terms at the top-right of the payoff function, from which their effort level is subtracted to give their overall utility outcome. In the case of rejection, they receive no boost, but suffer the same negative utility from their expenditure of effort.

Player *Re*’s base payoff from accepting or rejecting *Au*’s paper is 1. We also take *Re*’s effort costs into account. To incorporate these costs into the model, *Re*’s final payoff is affected by *Au*’s effort and *Re*’s own acceptance threshold. We assume that higher review standards are more costly for the reviewer than lower standards: this reflects the fact that higher review standards will tend to require that a reviewer investigates more questions about the paper’s details. Therefore, by choosing a strategy $r_{k}=\left( t_{m},d\right)$, *Re* incurs a cost of $\delta t_{m}$, where $\delta >0$ and $t_{m}\in T$ is *Re*’s chosen acceptance threshold. However, *Re* also gets a payoff bonus from accepted higher quality papers. This bonus is defined as $\delta e_{i}$, where $\delta >0$ and $e_{i}\in E$ is *Au*’s chosen effort level, such that $e_{i}\ge t_{m}$.

By combining the aforementioned incentives, we can define *Re*’s final payoffs with a payoff function $\pi _{Re}: E\times R\rightarrow {\mathbb {R}}$, which is such that

2 $$\begin{aligned} \forall \left( e_{i},r_{k}=\left( t_{m},d\right) \right) \in E\times R, \pi _{Re}\left( e_{i},r_{k}\right) =\left\{\begin{array}{ll}1+\delta \left( e_{i}-t_{m}\right) & \quad \text{ when } e_{i}\ge t_{m};\\ 1-\delta t_{m}& \quad \text{ otherwise }. \end{array}\right. \end{aligned}$$

Notice that the term $\delta \left( e_{i}-t_{m}\right)$ determines the degree to which *Re*’s utility is affected by the effort they save due to reviewing a paper that clearly exceeds their acceptance threshold.

We now consider the consequences of institutional environment on the structure of the game. We define players’ “community” as the people whose opinion constitutes the reputation of a player. For example, this could include the people reading notifications about new papers in a scientist’s sub-field, but also grant committees and appointment committees who are surveying the scientist’s CV. For reputation, we assume a simple model in which publishing in quality journals has a ratchet effect for players: among papers that can pass the desk at a high quality journal, a player can ratchet up their reputation (beyond their simple publication count) insofar as they can publish papers with above-average effort. This enables their research to “stand out” within their sub-field. Of course, in the real world, there are diminishing marginal utilities for new high effort publications: for instance, a single notable paper means more for a young scientist pursuing tenure than for a world-famous superstar scientist. On the other hand, top academics also face reputational pressures to maintain their reputation by continuing to publish at a high level. The strengths of these effects are not obvious. For our exploratory purposes and our focus on population level tendencies, we can simplify without obvious loss by assuming that each player has the same bonus for standing out from their peers.

Under open review, the community has access not only to *Au*’s accepted work, but also *Re*’s review of *Au*’s accepted paper. In this case, the community can take a more active role in evaluating the quality of the peer-review process and awarding a reputation bonus to authors and reviewers who promote above average effort standards. Under open review, *Au* and *Re* receive a reputation bonus from acceptance if *Re* adopts an acceptance threshold that is higher than the average of possible threshold levels. For *Au* and *Re*, this reputation bonus is defined as $\mu \mathrm {max}\left( t_{m}-{\overline{t}},0\right)$, where $\mu >0$, $t_{m}\in T$ is the acceptance threshold chosen by *Re*, and ${\overline{t}}=\frac{1}{6}\sum _{m\in \left\{ 1,...,6\right\} }t_{m}$ is the average of possible acceptance thresholds. This means that *Re* obtains a reputation bonus under open review if they are shown to have high requirements for author effort, relative to the average for reviewers in the sub-field. Similarly, *Au* receives a reputation bonus if they are seen to have successfully convinced a tougher-than-average reviewer to recommend their paper for acceptance. By separating the reputation boost from the article (represented by $\epsilon$) from the reputation bonuses from the review (represented by $\mu$) we can distinguish between circumstances where one of these reputation boosts is more important than the other.

By combining all the incentives together, we obtain each player’s final payoff function for the open review case:

3 $$\begin{aligned}&\forall \left( e_{i},r_{k}=\left( t_{m},d\right) \right) \in E\times R,\nonumber \\&\quad \pi ^{*}_{Au}\left( e_{i},r_{k}\right) =\left\{ \begin{array}{ll}1+\epsilon e_{i}-\beta e_{i}+\mu \mathrm {max}\left( t_{m}-{\overline{t}},0\right) &{} \quad \text{ if } e_{i}\ge t_{m};\\ 0-\beta e_{i}&{} \quad \text{ otherwise }. \end{array}\right. \end{aligned}$$

4 $$\begin{aligned}&\forall \left( e_{i},r_{k}=\left( t_{m},d\right) )\right) \in E\times R,\nonumber \\&\quad \pi _{Re}^{*}\left( e_{i},r_{k}\right) =\left\{ \begin{array}{ll}1+\delta \left( e_{i}-t_{m}\right) +\mu \mathrm {max}\left( t_{m}-{\overline{t}},0\right) &{}\quad \text{ if } e_{i}\ge t_{m};\\ 1-\delta t_{m}&{} \quad \text{ otherwise }. \end{array}\right. \end{aligned}$$

Formally, we thus have an almost identical situation to the double-blind review case. The only difference is that we incorporate the effects of greater transparency on the utilities of the authors and reviewers via the $\mu$ terms. In addition to being able to evaluate the interactive effects of different parameter values under a single reviewing system, we can also compare the effects of review systems on the evolutionary expansion of this base game in our full agent-based model, via asking whether values of $\mu$ greater than zero make a substantive difference, and to what degree any particular value of $\mu$ affects the simulation results. Consequently, from a fairly simple base game, we are able to answer a variety of questions about peer review systems’ effects on author effort in our model.^12^

### The Agent-Based Model

From an author’s perspective, reviewers are randomly assigned to them by editors. In choosing a research strategy (more or less effort put into a particular paper) authors must consider a wide range of information, including both their own experiences and those of their peers. To incorporate this situation into our model, we use an agent-based model in which authors can update their strategies. The model represents the interaction between a population of authors and a population of reviewers who are repeatedly matched to play the author-reviewer game. In our simulation game, we assume that ${\underline{t}}=0.2$. This parameter value gives a good range in which authors can choose strategies, while still reflecting our focus on well-run, good quality journals that almost always avoid publishing very low-effort papers via desk rejections. Each author is initially assigned one of the six effort levels from the set $E:=\left\{ {\underline{t}}+0.1,...,{\underline{t}}+0.6\right\} =\left\{ 0.3, 0.4, 0.5, 0.6, 0.7, 0.8\right\}$. Each reviewer is initially assigned one of the six acceptance thresholds from the set $T:=\left\{ {\underline{t}},{\underline{t}}+0.1,...,{\underline{t}}+0.5\right\} =\left\{ 0.2, 0.3, 0.4, 0.5, 0.6, 0.7\right\}$. This setup ensures that each author is accepted by at least two types of reviewers, to reflect the fact that authors are uncertain about what reviewers will require. Furthermore, assigning equal numbers of reviewers to each strategy is a reasonable choice, given that we have no reason to give an initial advantage to any particular effort level. We set both populations to contain 1800 agents.^13^ One advantage of this setting is that each author has a wide pool of peers who might adopt very different strategies. Across most disciplines, the number of contributing authors has grown from a relatively small community to a larger population in the last 60 years (Hamermesh, 2013).

In our model, players always have a discrete set of strategies to pick from. What if we allow them to pick from a continuous set of strategies? This setting would have some advantages for obtaining general analytical results about the Nash equilibria of the considered games. It could be an interesting topic for future research. However, this approach would have some disadvantages with respect to our current objectives. Firstly, a discrete set of strategies aides the realism of our model. We want to incorporate the imitation of successful peers by authors and reviewers, and it is unrealistic to suppose that imitators could make infinitely fine-grained discriminations between peers’ effort levels. It is also implausible that researchers have infinitely fine-grained control over their own effort levels. Secondly, we want to assume that researchers are boundedly rational, and so we use best response and logit choice protocols for strategy choices, as we detail below. In non-potential games, these approaches require a finite set of strategies. With logit response dynamics in particular, any meaningful convergence results within a finite number of simulation rounds can only be expected with a limited number of strategies in the initial population state.^14^ On the other hand, a model should also reflect the heterogeneity of the scientific community and its diverse choices of effort levels. If we just gave players two strategies – say, high and relatively low-effort – then we would be inadequately representing the extent to which authors and reviewers can adopt multiple increments of effort. We chose six strategies as a compromise between the advantages of greater and smaller numbers of possible strategies.

Additionally, while analytic proofs of equilibrium have their advantages, they have some limitations for studying policy changes, such as a journal’s change from double-blind reviewing to open review. An equilibrium analysis of the game is not sufficient to identify its evolutionarily and stochastically stable population states. By contrast, analytic methods can be used to study stochastically stable states in potential games, as well as evolutionarily stable states. Yet simulations have an additional advantage of revealing the convergence trajectories from the initial population states to the evolutionarily or stochastically stable states, and thus allows us to study convergence in our finite population games with multiple evolutionarily or stochastically stable states. In addition, simulations allow us to both study the evolutionary dynamics in games that do not have evolutionarily stable states and to evaluate the population convergence speed under different dynamics. Suppose that open review has a preferable evolutionarily or stochastically stable state. It is possible that convergence to a stable population state might take an extremely long time – potentially longer than the existence of the human species. If the benefits of the policy change take too long to manifest, then editors are likely to revert to the more familiar system. Additionally, if they revert or the benefits do not manifest in a practically relevant period of time, then editors will have made the adjustment costs without obtaining the benefits of the preferable system. A simulation-based approach has the advantage that we can obtain information about both the evolutionarily and stochastically stable properties of the model *and* its convergence tendencies.

In the initial population state, each strategy and effort level is assigned to 300 agents. This is done to eliminate any artificial bias which may act in favour of certain types of agents. We use a probabilistic strategy revision procedure: in every round of the game, there is a 0.122 probability of each agent being assigned a strategy revision opportunity. This number means that, on average, around 219 agents have the opportunity to revise strategies in each round. The strategy revision procedure is imitative. Once the agent has an opportunity to revise their strategy, the agent compiles a list of *n* candidates which includes $n-1$ randomly picked agents and the strategy revising agent. Thus, strategies which are used by a larger number of agents are more likely to be used by the randomly chosen candidates than the less popular strategies. This reflects the tendency to follow the crowd. One reason why authors might adopt this behaviour is that the popularity of a strategy might be due to its past evolutionary success.

Each candidate plays the agent-reviewer game against every type of opponent, and the strategy revising agent observes the average payoff obtained by each candidate. The strategy revising agent thus obtains a candidate record $\left( s,{\overline{\pi }}\right)$ – a $n\times 2$ matrix where the first column $s:=\left\{ s_{h}\right\} ^{n}_{h=1}$ is the strategy record, the second column ${\overline{\pi }}:=\left\{ {\overline{\pi }}_{h}\right\} ^{n}_{h=1}$ is the average payoff record, and the *h*th row of the record is a pair $\left( s_{h},{\overline{\pi }}_{h}\right)$ that describes the strategy played and the average payoff obtained at revising agent’s *h*th observation. If a strategy revising agent chooses from finite set of strategies $S=\left( 1,...,z\right)$, where $z\ge 2$, and uses strategy $b\in S$, then the best response protocol defines the probability of agent switching to strategy $c\in S$ as

5 $$\begin{aligned} \text{ if } \left[ {\overline{\pi }}_{h}=\mathrm {max}_{h'}{\overline{\pi }}_{h'}\Rightarrow s_{h}=c\right] , \text{ then } \sigma _{bc}\left( s,{\overline{\pi }}\right) =1. \end{aligned}$$

If a player’s strategy *b* is in the set of candidate strategies with the highest average payoff record, then the player sticks to strategy *b*. If the set of strategies with the highest average payoff record contains multiple strategies but does not contain a player’s strategy *b*, then the player makes a uniformly randomized choice among the strategies in that set.

The logit choice protocol defines the probability of agent switching to strategy $c\in S$ as

6 $$\begin{aligned} \sigma _{bc}\left( s,{\overline{\pi }}\right) =\frac{\sum _{h:s_{h}=c}\mathrm {exp}\left( \eta ^{-1}{\overline{\pi }}_{h}\right) }{\sum _{v\in S}\sum _{h:s_{h}=v}\mathrm {exp}\left( \eta ^{-1}{\overline{\pi }}_{h}\right) }. \end{aligned}$$

$\eta$ is the noise factor that we use to represent the bounded rationality of players, who might plausibly make mistakes when trying to identify an optimal strategy. For example, an author might think that switching to a new effort level will increase their payoff, based on their peer assessments, even when this switch is not warranted by their evidence. For candidate selection, we assume that $n=31$. This is an approximately realistic figure: researchers can assess the effort levels of their peers, but not too many of them. For example, by reading an article in depth, one can obtain a sense of how much effort the authors put into the article, but one cannot do this for a large number of articles in a given time-span. Furthermore, less direct sources of information about a scientist’s research quality (conferences, seminars, and other networking events) are often unreliable, and it is hard to determine their weighting for professional reputations. Therefore, we do not incorporate them into this version of our model – a model incorporating (potentially severe) uncertainty about effort levels is an exciting direction for future research.

We set a low logit noise level $\eta =0.044$.^15^ In addition, for one set of simulations, we assume that each player may randomly switch to any strategy. The probability of this event is set to 0.008, which represents the potential influences on strategy selection that are not included in our model: funding challenges or successes, authors entering a new and unfamiliar field, reviewers raising their methodological standards after reading a book or article, and so on. Thus, we allow for both strategy switches based on mistakes and strategy switches based on factors beyond those that we explicitly include in our model. By including both sources of randomness and separating them, our model can flexibly represent a wide range of scenarios. These stochastic elements in our model thereby incorporate unpredictable and complex factors which introduce multifarious uncertainties into the review process.

### The Simulation Process

For simulations, we used the ABED simulation package for NetLogo developed by Izquierdo et al. (2019). Each simulation was run for 13,000 rounds. This large number of rounds helps to identify the long-run tendencies of a particular game. In the simulations, we assumed some of the parameter values in the author-reviewer game to be fixed. The value of $\beta$ was set to 0.1. We used three values for parameter $\epsilon$ – 0, 0.1 and 0.2. For each value of $\epsilon$, we used three values of parameter $\delta$ – 0.1, 0.2 and 0.3, and five values of parameter $\mu$ – 0, 0.2, 0.4, 0.6, and 0.8. Therefore, by keeping costs fixed, we can isolate the impact of the institutional environment and how it shapes reputation effects.

Prior to the simulations, for each combination of the values of $\epsilon$, $\delta$, and $\mu$ that we considered, we calculated the related payoff matrix by a Python 3 script, according to the previous Eqs. 1 to 4. A total of 45 matrices-all bearing dimensions $6\times 6$ – were computed. See Tables 1, 2, and 3 for examples. For each payoff matrix, we initially verified if the matrix showed potential game features. Using a criterion provided by Sandholm (2010b), we checked for each entry if:

7 $$a_{ij}+\frac{1}{36}\sum _{i,\ j}a_{ij}-\frac{1}{6}\Bigg (\sum _{i}a_{ij}+\sum _{j}a_{ij}\Bigg )=b_{ij}+\frac{1}{36}\sum _{i,\ j}b_{ij}-\frac{1}{6}\Bigg (\sum _{i}b_{ij}+\sum _{j}b_{ij}\Bigg ),$$

where $a_{ij}$ and $b_{ij}$ represent players’ payoffs. As we discussed earlier, none of the matrices is characterised by this property, nor satisfies the minimum conditions to be considered a representation of a near-potential game (Lahkar & Riedel, 2015). Additionally, using the Python library Nashpy (Knight & Campbell, 2018), we investigated the existence and number of Nash equilibria in pure strategies in every payoff matrix. We give the number of Nash equilibria for various values of $\delta$ and $\mu$ in Tables 4, 5 and 6.

**Table 1:** Examples of payoff matrices for $\epsilon =0.2$

		Reviewer					
		$r=\left( 0.2,d\right)$	$r=\left( 0.3,d\right)$	$r=\left( 0.4,d\right)$	$r=\left( 0.5,d\right)$	$r=\left( 0.6,d\right)$	$r=\left( 0.7,d\right)$
(a) Payoff matrix computed for $(\delta ,\mu )=(0.2,0)$							
Author	$e=0.3$	(1.03, 1.02)	(1.03, 1.00)	$(-\,0.03,0.92)$	$(-\,0.03,0.90)$	$(-\,0.03,0.88)$	$(-\,0.03,0.86)$
	$e=0.4$	(1.04, 1.04)	(1.04, 1.02)	(1.04, 1.00)	$(-\,0.04,0.90)$	$(-\,0.04,0.88)$	$(-\,0.04,0.86)$
	$e=0.5$	(1.05, 1.06)	(1.05, 1.04)	(1.05, 1.02)	(1.05, 1.00)	$(-\,0.05,0.88)$	$(-\,0.05,0.86)$
	$e=0.6$	(1.06, 1.08)	(1.06, 1.06)	(1.06, 1.04)	(1.06, 1.02)	(1.06, 1.00)	$(-\,0.06,0.86)$
	$e=0.7$	(1.07, 1.10)	(1.07, 1.08)	(1.07, 1.06)	(1.07, 1.04)	(1.07, 1.02)	(1.07, 1.00)
	$e=0.8$	(**1.08**, **1.12**)	(1.08, 1.10)	(1.08, 1.08)	(1.08, 1.06)	(1.08, 1.04)	(1.08, 1.02)
(b) Payoff matrix computed for $(\delta ,\mu )=(0.2,0.2)$							
Author	$e=0.3$	(1.03, 1.02)	(1.03, 1.00)	$(-\,0.03,0.92)$	$(-\,0.03,0.9)$	$(-\,0.03,0.88)$	$(-\,0.03,0.86)$
	$e=0.4$	(1.04, 1.04)	(1.04, 1.02)	(1.04, 1.00)	$(-\,0.04,0.9)$	$(-\,0.04,0.88)$	$(-\,0.04,0.86)$
	$e=0.5$	(1.05, 1.06)	(1.05, 1.04)	(1.05, 1.02)	(1.06, 1.01)	$(-\,0.05,0.88)$	$(-\,0.05,0.86)$
	$e=0.6$	(1.06, 1.08)	(1.06, 1.06)	(1.06, 1.04)	(1.07, 1.03)	(1.09, 1.03)	$(-\,0.06,0.86)$
	$e=0.7$	(1.07, 1.10)	(1.07, 1.08)	(1.07, 1.06)	(1.08, 1.05)	(1.10, 1.05)	(1.12, 1.05)
	$e=0.8$	(**1.08**, **1.12**)	(1.08, 1.10)	(1.08, 1.08)	(1.09, 1.07)	(1.11, 1.07)	(1.13, 1.07)
(c) Payoff matrix computed for $(\delta ,\mu )=(0.2,0.4)$							
Author	$e=0.3$	(1.03, 1.02)	(1.03, 1.00)	$(-\,0.03,0.92)$	$(-\,0.03,0.90)$	$(-\,0.03,0.88)$	$(-\,0.03,0.86)$
	$e=0.4$	(1.04, 1.04)	(1.04, 1.02)	(1.04, 1.00)	$(-\,0.04,0.9)$	$(-\,0.04,0.88)$	$(-\,0.04,0.86)$
	$e=0.5$	(1.05, 1.06)	(1.05, 1.04)	(1.05, 1.02)	(1.07, 1.02)	$(-\,0.05,0.88)$	$(-\,0.05,0.86)$
	$e=0.6$	(1.06, 1.08)	(1.06, 1.06)	(1.06, 1.04)	(1.08, 1.04)	(1.12, 1.06)	$(-\,0.06,0.86)$
	$e=0.7$	(1.07, 1.10)	(1.07, 1.08)	(1.07, 1.06)	(1.09, 1.06)	(1.13, 1.08)	(1.17, 1.10)
	$e=0.8$	(**1.08**, **1.12**)	(1.08, 1.10)	(1.08, 1.08)	(1.10, 1.08)	(1.14, 1.10)	(**1.18**, **1.12**)

Matrix (1a) is defined for a double-blind system and it is characterized by having one Nash equilibrium in pure strategies, whereas matrices (1b) and (1c) are calculated for an open review system with $\mu =0.2$ and $\mu =0.4,$ and respectively show one and two Nash equilibria in pure strategies. Nash equilibria are marked by bold text in each matrix

**Table 2:** Likewise Table 1, examples of payoff matrices are reported for $\epsilon =0.1$

		Reviewer					
		$r=\left( 0.2,d\right)$	$r=\left( 0.3,d\right)$	$r=\left( 0.4,d\right)$	$r=\left( 0.5,d\right)$	$r=\left( 0.6,d\right)$	$r=\left( 0.7,d\right)$
(a) Payoff matrix computed for $(\delta ,\mu )=(0.1,0)$							
Author	$e=0.3$	(**1.00**, **1.01**)	(1.00, 1.00)	$(-\,0.03,0.96)$	$(-\,0.03,0.95)$	$(-\,0.03,0.94)$	$(-\,0.03,0.93)$
	$e=0.4$	(**1.00**, **1.02**)	(1.00, 1.01)	(1.00, 1.00)	$(-\,0.04,0.95)$	$(-\,0.04,0.94)$	$(-\,0.04,0.93)$
	$e=0.5$	(**1.00**, **1.03**)	(1.00, 1.02)	(1.00, 1.01)	(1.00, 1.00)	$(-\,0.05,0.94)$	$(-\,0.05,0.93)$
	$e=0.6$	(**1.00**, **1.04**)	(1.00, 1.03)	(1.00, 1.02)	(1.00, 1.01)	(1.00, 1.00)	$(-\,0.06,0.93)$
	$e=0.7$	(**1.00**, **1.05**)	(1.00, 1.04)	(1.00, 1.03)	(1.00, 1.02)	(1.00, 1.01)	(1.00, 1.00)
	$e=0.8$	(**1.00**, **1.06**)	(1.00, 1.05)	(1.00, 1.04)	(1.00, 1.03)	(1.00, 1.02)	(1.00, 1.01)
(b) Payoff matrix computed for $(\delta ,\mu )=(0.1,0.2)$							
Author	$e=0.3$	(**1.00**, **1.01**)	(1.00, 1.00)	$(-\,0.03,0.96)$	$(-\,0.03,0.95)$	$(-\,0.03,0.94)$	$(-\,0.03,0.93)$
	$e=0.4$	(**1.00**, **1.02**)	(1.00, 1.01)	(1.00, 1.00)	$(-\,0.04,0.95)$	$(-\,0.04,0.94)$	$(-\,0.04,0.93)$
	$e=0.5$	(**1.00**, **1.03**)	(1.00, 1.02)	(1.00, 1.01)	(1.01, 1.01)	$(-\,0.05,0.94)$	$(-\,0.05,0.93)$
	$e=0.6$	(**1.00**, **1.04**)	(1.00, 1.03)	(1.00, 1.02)	(1.01, 1.02)	(1.03, 1.03)	$(-\,0.06,0.93)$
	$e=0.7$	(**1.00**, **1.05**)	(1.00, 1.04)	(1.00, 1.03)	(1.01, 1.03)	(1.03, 1.04)	(**1.05**, **1.05**)
	$e=0.8$	(**1.00**, **1.06**)	(1.00, 1.05)	(1.00, 1.04)	(1.01, 1.04)	(1.03, 1.05)	(**1.05**, **1.06**)
(c) Payoff matrix computed for $(\delta ,\mu )=(0.1,0.4)$							
Author	$e=0.3$	(**1.00**, **1.01**)	(1.00, 1.00)	$(-\,0.03,0.96)$	$(-\,0.03,0.95)$	$(-\,0.03,0.94)$	$(-\,0.03,0.93)$
	$e=0.4$	(**1.00**, **1.02**)	(1.00, 1.01)	(1.00, 1.00)	$(-\,0.04,0.95)$	$(-\,0.04,0.94)$	$(-\,0.04,0.93)$
	$e=0.5$	(**1.00**, **1.03**)	(1.00, 1.02)	(1.00, 1.01)	(1.02, 1.02)	$(-\,0.05,0.94)$	$(-\,0.05,0.93)$
	$e=0.6$	(1.00, 1.04)	(1.00, 1.03)	(1.00, 1.02)	(1.02, 1.03)	(**1.06**, **1.06**)	$(-\,0.06,0.93)$
	$e=0.7$	(1.00, 1.05)	(1.00, 1.04)	(1.00, 1.03)	(1.02, 1.04)	(1.06, 1.07)	(**1.10**, **1.10**)
	$e=0.8$	(1.00, 1.06)	(1.00, 1.05)	(1.00, 1.04)	(1.02, 1.05)	(1.06, 1.08)	(**1.10**, **1.11**)

Matrix (2a) is defined for a double-blind system (six Nash equilibria in pure strategies), whereas matrices (2b) and (2c) are computed for an open review system, respectively showing eight and six Nash equilibria in pure strategies. Again, bold text distinguishes Nash equilibria within each matrix

**Table 3:** Similarly to Tables 1 and 2, examples of payoff matrices are shown for $\epsilon =0$

		Reviewer					
		$r=\left( 0.2,d\right)$	$r=\left( 0.3,d\right)$	$r=\left( 0.4,d\right)$	$r=\left( 0.5,d\right)$	$r=\left( 0.6,d\right)$	$r=\left( 0.7,d\right)$
(a) Payoff matrix computed for $(\delta ,\mu )=(0.2,0)$							
Author	$e=0.3$	(**0.97**, **1.02**)	(0.97, 1.00)	$(-\,0.03,0.92)$	$(-\,0.03,0.90)$	$(-\,0.03,0.88)$	$(-\,0.03,0.86)$
	$e=0.4$	(0.96, 1.04)	(0.96, 1.02)	(0.96, 1.00)	$(-\,0.04,0.90)$	$(-\,0.04,0.88)$	$(-\,0.04,0.86)$
	$e=0.5$	(0.95, 1.06)	(0.95, 1.04)	(0.95, 1.02)	(0.95, 1.00)	$(-\,0.05,0.88)$	$(-\,0.05,0.86)$
	$e=0.6$	(0.94, 1.08)	(0.94, 1.06)	(0.94, 1.04)	(0.94, 1.02)	(0.94, 1.00)	$(-\,0.06,0.86)$
	$e=0.7$	(0.93, 1.10)	(0.93, 1.08)	(0.93, 1.06)	(0.93, 1.04)	(0.93, 1.02)	(0.93, 1.00)
	$e=0.8$	(0.92, 1.12)	(0.92, 1.10)	(0.92, 1.08)	(0.92, 1.06)	(0.92, 1.04)	(0.92, 1.02)
(b) Payoff matrix computed for $(\delta ,\mu )=(0.2,0.2)$							
Author	$e=0.3$	(**0.97**, **1.02**)	(0.97, 1.00)	$(-\,0.03,0.92)$	$(-\,0.03,0.9)$	$(-\,0.03,0.88)$	$(-\,0.03,0.86)$
	$e=0.4$	(0.96, 1.04)	(0.96, 1.02)	(0.96, 1.00)	$(-\,0.04,0.9)$	$(-\,0.04,0.88)$	$(-\,0.04,0.86)$
	$e=0.5$	(0.95, 1.06)	(0.95, 1.04)	(0.95, 1.02)	(0.96, 1.01)	$(-\,0.05,0.88)$	$(-\,0.05,0.86)$
	$e=0.6$	(0.94, 1.08)	(0.94, 1.06)	(0.94, 1.04)	(0.95, 1.03)	(0.97, 1.03)	$(-\,0.06,0.86)$
	$e=0.7$	(0.93, 1.10)	(0.93, 1.08)	(0.93, 1.06)	(0.94, 1.05)	(0.96, 1.05)	(0.98, 1.05)
	$e=0.8$	(0.92, 1.12)	(0.92, 1.10)	(0.92, 1.08)	(0.93, 1.07)	(0.95, 1.07)	(0.97, 1.07)
(c) Payoff matrix computed for $(\delta ,\mu )=(0.2,0.4)$							
Author	$e=0.3$	(**0.97**, **1.02**)	(0.97, 1.00)	$(-\,0.03,0.92)$	$(-\,0.03,0.9)$	$(-\,0.03,0.88)$	$(-\,0.03,0.86)$
	$e=0.4$	(0.96, 1.04)	(0.96, 1.02)	(0.96, 1.00)	$(-\,0.04,0.90)$	$(-\,0.04,0.88)$	$(-\,0.04,0.86)$
	$e=0.5$	(0.95, 1.06)	(0.95, 1.04)	(0.95, 1.02)	(0.97, 1.02)	$(-\,0.05,0.88)$	$(-\,0.05,0.86)$
	$e=0.6$	(0.94, 1.08)	(0.94, 1.06)	(0.94, 1.04)	(0.96, 1.04)	(1.00, 1.06)	$(-\,0.06,0.86)$
	$e=0.7$	(0.93, 1.10)	(0.93, 1.08)	(0.93, 1.06)	(0.95, 1.06)	(0.99, 1.08)	(**1.03**, **1.10**)
	$e=0.8$	(0.92, 1.12)	(0.92, 1.10)	(0.92, 1.08)	(0.94, 1.08)	(0.98, 1.10)	(1.02, 1.12)

Matrix (3a) is defined for a double-blind system with one Nash equilibrium in pure strategies, whereas matrices (3b) and (3c) are calculated for an open review system: one and two Nash equilibria in pure strategies, respectively. Bold text distinguishes Nash equilibria within each matrix

**Table 4:** Number of pure-strategy Nash equilibria in games computed for $\epsilon =0$ at varying levels of $\delta$ and $\mu$

$\delta$	Double-blind	Open review			
	$$\mu $$				
	$\mu =0$	$\mu =0.2$	$\mu =0.4$	$\mu =0.6$	$\mu =0.8$
$\delta =0.1$	1	2	3	4	4
$\delta =0.2$	1	1	2	3	3
$\delta =0.3$	1	1	1	2	3

**Table 5:** Number of pure-strategy Nash equilibria in games computed for $\epsilon =0.1$ at varying levels of $\delta$ and $\mu$

$\delta$	Double-blind	Open review			
	$$\mu $$				
	$\mu =0$	$\mu =0.2$	$\mu =0.4$	$\mu =0.6$	$\mu =0.8$
$\delta =0.1$	6	8	6	7	6
$\delta =0.2$	6	6	8	6	6
$\delta =0.3$	6	6	6	8	7

**Table 6:** Number of pure-strategy Nash equilibria in games computed for $\epsilon =0.2$ at varying levels of $\delta$ and $\mu$

$\delta$	Double-blind	Open review			
	$$\mu $$				
	$\mu =0$	$\mu =0.2$	$\mu =0.4$	$\mu =0.6$	$\mu =0.8$
$\delta =0.1$	1	2	1	1	1
$\delta =0.2$	1	1	2	1	1
$\delta =0.3$	1	1	1	2	1

After the Nash equilibria check, we ran simulations for the set number of rounds for each payoff matrix, and we assessed a discrete probability distribution *P* for the 6 strategies we considered. In all simulations, the final goal was to compute the expected value of authors’ effort levels as

8 $$\begin{aligned} {\mathbb {E}}[E]=\sum _{i=1}^6e_ip_i \end{aligned}$$

and, similarly, the related expected value of reviewers’ threshold levels.

We report the expected values for the logit dynamics case with noise and with $\epsilon =\{0,0.1,0.2\}$ for authors in Tables 7, 9, and 11. The corresponding data for reviewers is given in Tables 8, 10, and 12.

**Table 7:** Expected values of effort levels (authors) computed for $\epsilon =0$ over simulations lasting 13,000 rounds with logit dynamics and noise

$\delta$	Double-blind	Open review			
	$$\mu $$				
	$\mu =0$	$\mu =0.2$	$\mu =0.4$	$\mu =0.6$	$\mu =0.8$
$\delta =0.1$	0.675	0.697	0.709	0.714	0.718
$\delta =0.2$	0.554	0.578	0.707	0.714	0.716
$\delta =0.3$	0.484	0.498	0.524	0.602	0.715

**Table 8:** Expected values of threshold levels (reviewers) computed for $\epsilon =0$ over simulations lasting 13,000 rounds with logit dynamics and noise

$\delta$	Double-blind	Open review			
	$$\mu $$				
	$\mu =0$	$\mu =0.2$	$\mu =0.4$	$\mu =0.6$	$\mu =0.8$
$\delta =0.1$	0.281	0.359	0.639	0.671	0.680
$\delta =0.2$	0.232	0.239	0.377	0.647	0.672
$\delta =0.3$	0.222	0.224	0.226	0.231	0.675

**Table 9:** Expected values of effort levels (authors) computed for $\epsilon =0.1$ over simulations lasting 13,000 rounds with logit dynamics and noise

$\delta$	Double-blind	Open review			
	$$\mu $$				
	$\mu =0$	$\mu =0.2$	$\mu =0.4$	$\mu =0.6$	$\mu =0.8$
$\delta =0.1$	0.716	0.730	0.741	0.743	0.748
$\delta =0.2$	0.691	0.702	0.741	0.749	0.757
$\delta =0.3$	0.668	0.690	0.693	0.723	0.745

**Table 10:** Expected values of threshold levels (reviewers) computed for $\epsilon =0.1$ over simulations lasting 13,000 rounds with logit dynamics and noise

$\delta$	Double-blind	Open review			
	$$\mu $$				
	$\mu =0$	$\mu =0.2$	$\mu =0.4$	$\mu =0.6$	$\mu =0.8$
$\delta =0.1$	0.278	0.462	0.649	0.671	0.679
$\delta =0.2$	0.245	0.249	0.367	0.647	0.673
$\delta =0.3$	0.226	0.229	0.236	0.275	0.656

**Table 11:** Expected values of effort levels (authors) computed for $\epsilon =0.2$ over simulations lasting 13,000 rounds with logit dynamics and noise

$\delta$	Double-blind	Open review			
	$$\mu $$				
	$\mu =0$	$\mu =0.2$	$\mu =0.4$	$\mu =0.6$	$\mu =0.8$
$\delta =0.1$	0.760	0.767	0.771	0.778	0.781
$\delta =0.2$	0.754	0.756	0.775	0.779	0.785
$\delta =0.3$	0.750	0.751	0.753	0.771	0.782

**Table 12:** Expected values of threshold levels (reviewers) computed for $\epsilon =0.2$ over simulations lasting 13,000 rounds with logit dynamics and noise

$\delta$	Double-blind	Open review			
	$$\mu $$				
	$\mu =0$	$\mu =0.2$	$\mu =0.4$	$\mu =0.6$	$\mu =0.8$
$\delta =0.1$	0.282	0.418	0.648	0.672	0.680
$\delta =0.2$	0.241	0.253	0.367	0.655	0.671
$\delta =0.3$	0.227	0.232	0.239	0.402	0.656

**Fig. 1:**
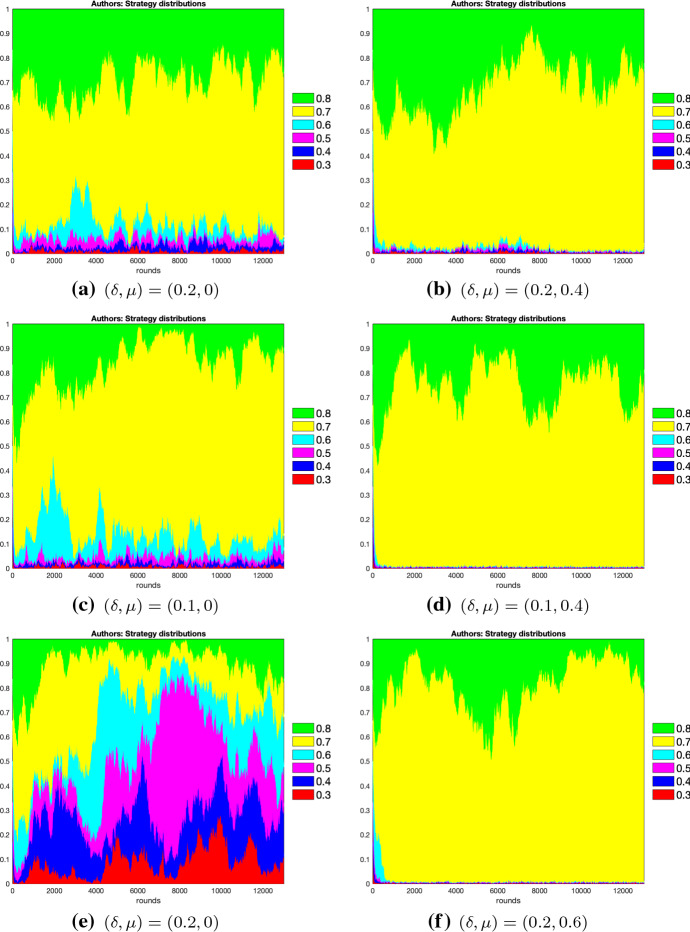
Strategy distributions for authors under logit dynamics with noise. On left panels, figures representing interactions under double-blind system are shown, whereas on right panels we reproduce examples under open review. Strategies percentages (Y-axis) are pictured against rounds (X-axis). From the top to the bottom of the figures, we display authors’ strategy distributions with logit dynamics and noise for $\epsilon =0.2$ (**a** and **b**), for $\epsilon =0.1$ (**c** and **d**) and for $\epsilon =0$ (**e** and **f**). The highest author effort level is 0.8, with its related strategy coloured in green, and the lowest is 0.3, with its related strategy coloured in red

**Fig. 2:**
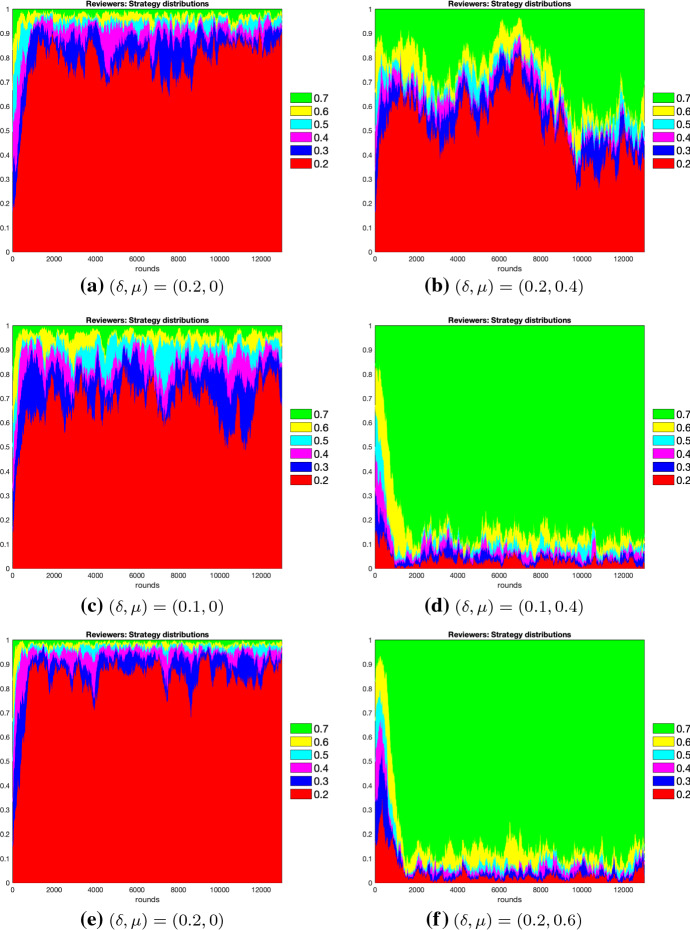
Strategy distributions for reviewers under logit dynamics with noise. Left and right panels mirror for reviewers what is presented for authors in Fig. 1. Similarly, from the top to the bottom we display reviewers’ strategy distributions with logit dynamics and noise for $\epsilon =0.2$ (**a** and **b**), for $\epsilon =0.1$ (**c** and **d**) and for $\epsilon =0$ (**e** and **f**). Numbers and colours in the graphs have the same meanings as in Fig. 1, with the highest reviewer threshold level equal to 0.7, and the lowest equal to 0.2

**Fig. 3:**
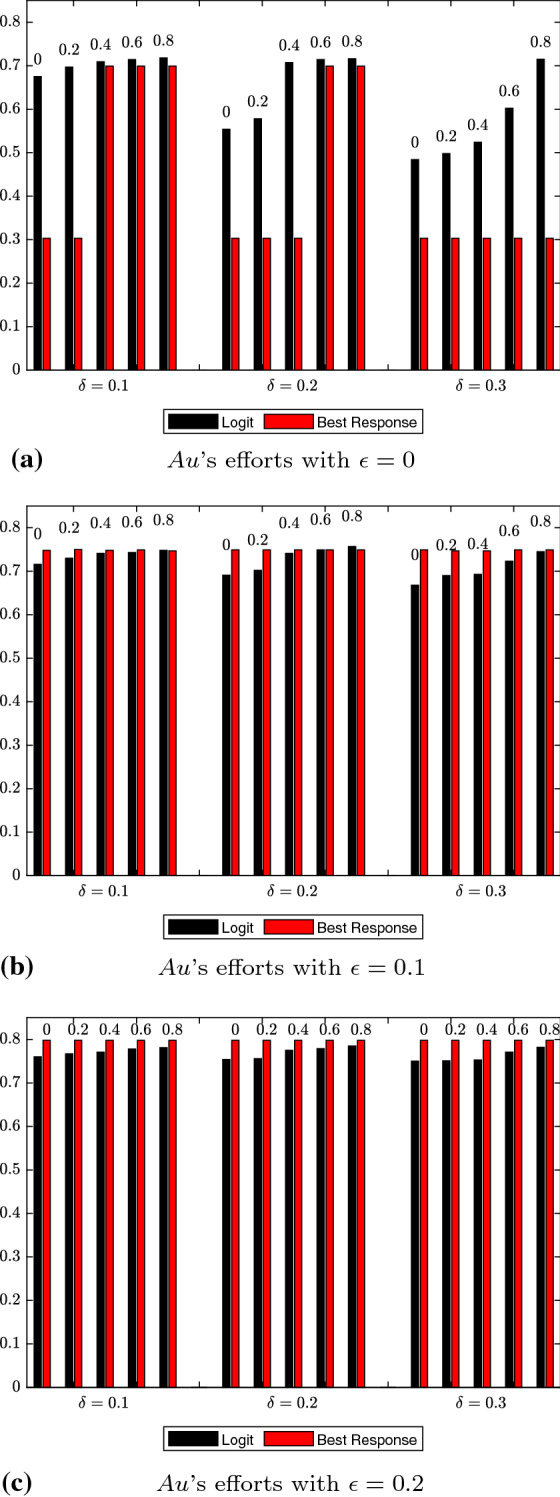
Comparison of expected values of effort levels under logit dynamics with noise and best response dynamics with noise for authors. Results at varying levels of $\epsilon$ are displayed in (**a**, **b** and **c**). In each subfigure, the numbers atop each bar represent $\mu$ values. $\delta$ values are reported on the X-axis, whereas expected values of effort levels are shown on the Y-axis. The data shown come from Tables 7, 9, 11, 25, 27 and 29

**Fig. 4:**
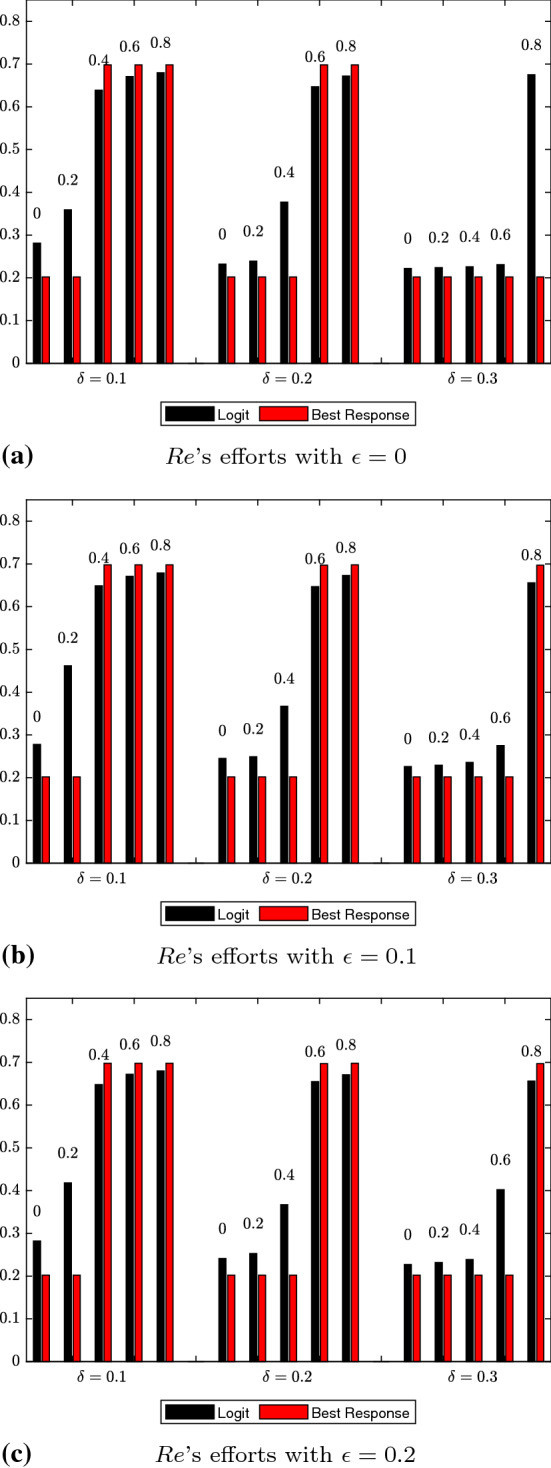
Comparison of expected values of threshold levels under logit dynamics with noise and best response dynamics with noise for reviewers. Results at varying levels of $\epsilon$ are displayed in (**a**, **b**, **c**). Like Fig. 3, we report combinations of $\delta$ and $\mu$ values in each subfigure. The data shown come from Tables 8, 10, 12, 26, 28 and 30

**Fig. 5:**
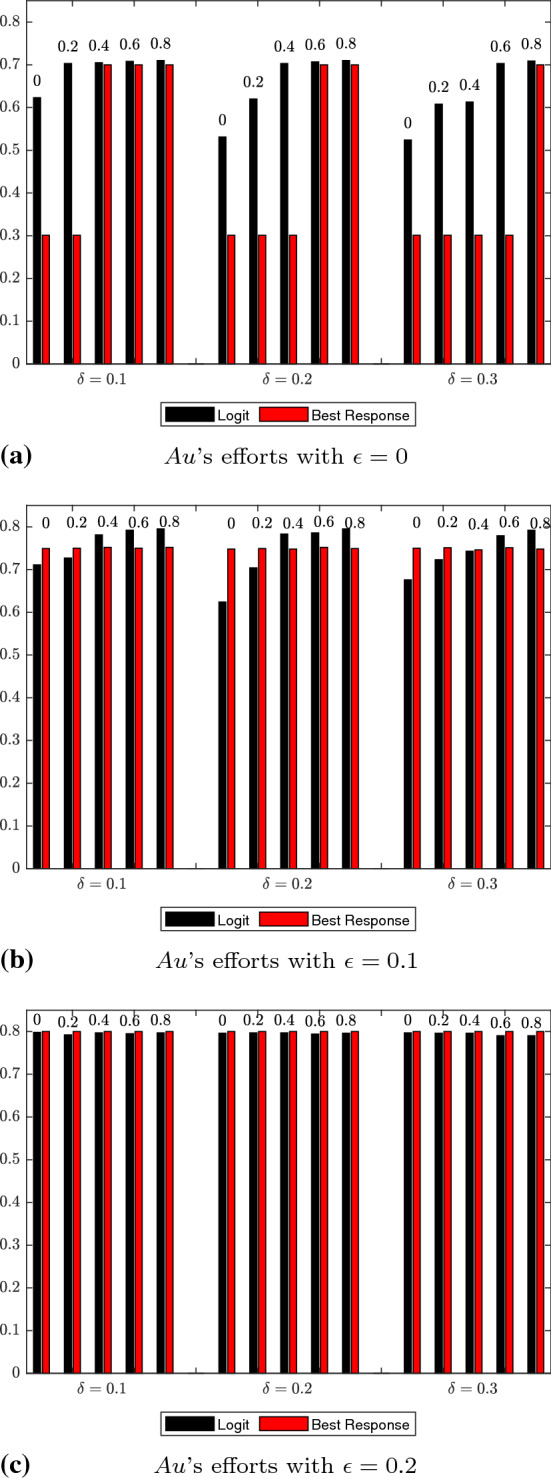
Comparison of expected values of effort levels under logit dynamics without noise and best response dynamics without noise for authors. Results at varying levels of $\epsilon$ are displayed in (**a**, **b** and **c**). Like Figs. 3 and 4, we report combinations of $\delta$ and $\mu$ values in each subfigure. The data shown come from Tables 13, 15, 17, 19, 21 and 23

**Fig. 6:**
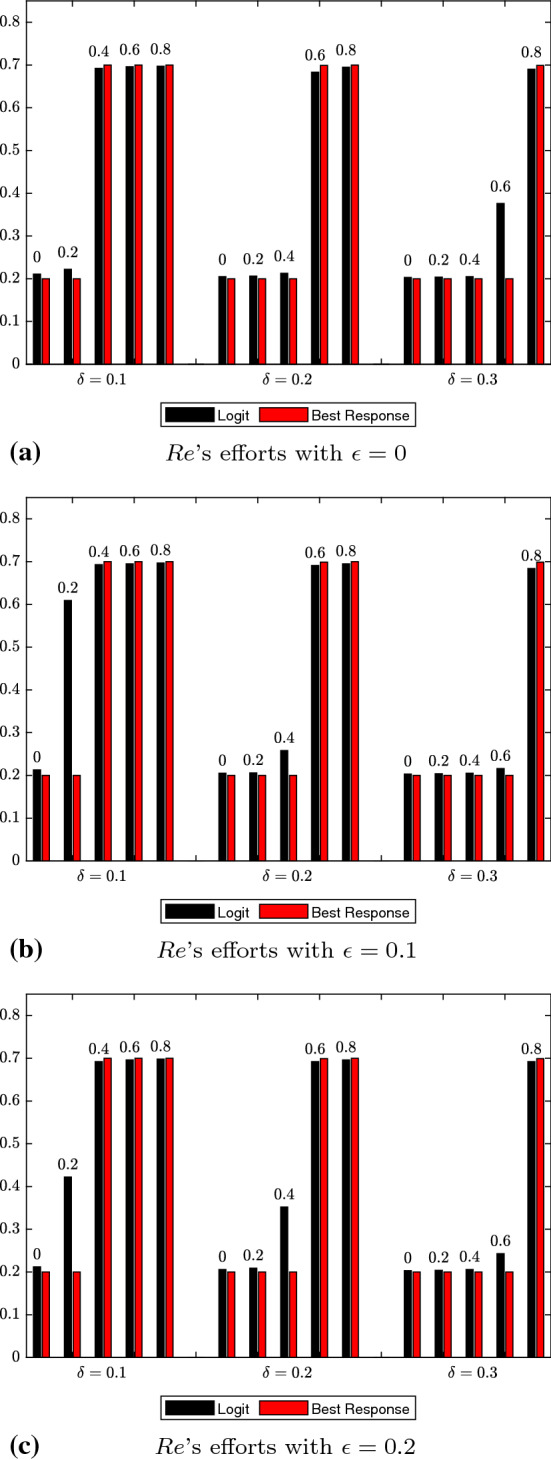
Comparison of expected values of threshold levels under logit dynamics without noise and best response dynamics without noise for reviewers. Results at varying levels of $\epsilon$ are displayed in (**a**, **b** and **c**). Like Figs. 3 to 5, we report combinations of $\delta$ and $\mu$ values in each subfigure. The data shown come from Tables 14, 16, 18, 20, 22 and 24

For the logit dynamics case with noise, we provide graphical comparisons of the strategy distributions for authors and reviewers in Figs. 1 and 2. Additionally, in Figs. 3 and 4 we show how the expected values of effort levels for authors and threshold levels for reviewers vary, both under logit dynamics and best response dynamics with noise (for the latter, see Tables 25, 26, 27, 28, 29 and 30). For the sake of comparison, we also report the expected values for the logit dynamics case without noise in Tables 13, 14, 15, 16, 17, and 18 and for the best response case without noise in Tables 19, 20, 21, 22, 23 and 24. Finally, in Figs. 5 and 6 we offer a graphical comparison of the expected values of effort levels for authors and threshold levels for reviewers, under both logit dynamics and best response dynamics without noise.

**Table 13:** Expected values of effort levels (authors) computed for $\epsilon =0$ over simulations lasting 13,000 rounds with logit dynamics and no noise

$\delta$	Double-blind	Open review			
	$$\mu $$				
	$\mu =0$	$\mu =0.2$	$\mu =0.4$	$\mu =0.6$	$\mu =0.8$
$\delta =0.1$	0.623	0.703	0.705	0.708	0.710
$\delta =0.2$	0.531	0.620	0.703	0.707	0.710
$\delta =0.3$	0.524	0.608	0.613	0.703	0.709

**Table 14:** Expected values of threshold levels (reviewers) computed for $\epsilon =0$ over simulations lasting 13,000 rounds with logit dynamics and no noise

$\delta$	Double-blind	Open review			
	$$\mu $$				
	$\mu =0$	$\mu =0.2$	$\mu =0.4$	$\mu =0.6$	$\mu =0.8$
$\delta =0.1$	0.211	0.222	0.692	0.696	0.697
$\delta =0.2$	0.205	0.206	0.213	0.683	0.695
$\delta =0.3$	0.203	0.204	0.205	0.376	0.690

**Table 15:** Expected values of effort levels (authors) computed for $\epsilon =0.1$ over simulations lasting 13,000 rounds with logit dynamics and no noise

$\delta$	Double-blind	Open review			
	$$\mu $$				
	$\mu =0$	$\mu =0.2$	$\mu =0.4$	$\mu =0.6$	$\mu =0.8$
$\delta =0.1$	0.711	0.727	0.781	0.792	0.795
$\delta =0.2$	0.624	0.704	0.783	0.786	0.795
$\delta =0.3$	0.676	0.723	0.743	0.779	0.792

**Table 16:** Expected values of threshold levels (reviewers) computed for $\epsilon =0.1$ over simulations lasting 13,000 rounds with logit dynamics and no noise

$\delta$	Double-blind	Open review			
	*μ*				
	$\mu =0$	$\mu =0.2$	$\mu =0.4$	$\mu =0.6$	$\mu =0.8$
$\delta =0.1$	0.213	0.609	0.693	0.695	0.697
$\delta =0.2$	0.205	0.206	0.258	0.691	0.695
$\delta =0.3$	0.203	0.204	0.205	0.216	0.684

**Table 17:** Expected values of effort levels (authors) computed for $\epsilon =0.2$ over simulations lasting 13,000 rounds with logit dynamics and no noise

$\delta$	Double-blind	Open review			
	$$\mu $$				
	$\mu =0$	$\mu =0.2$	$\mu =0.4$	$\mu =0.6$	$\mu =0.8$
$\delta =0.1$	0.798	0.792	0.797	0.795	0.797
$\delta =0.2$	0.796	0.797	0.797	0.794	0.796
$\delta =0.3$	0.797	0.796	0.796	0.790	0.790

**Table 18:** Expected values of threshold levels (reviewers) computed for $\epsilon =0.2$ over simulations lasting 13,000 rounds with logit dynamics and no noise

$\delta$	Double-blind	Open review			
	$$\mu $$				
	$\mu =0$	$\mu =0.2$	$\mu =0.4$	$\mu =0.6$	$\mu =0.8$
$\delta =0.1$	0.212	0.422	0.692	0.696	0.698
$\delta =0.2$	0.206	0.209	0.352	0.692	0.696
$\delta =0.3$	0.203	0.204	0.206	0.243	0.692

**Table 19:** Expected values of effort levels (authors) computed for $\epsilon =0$ over simulations lasting 13,000 rounds with best response dynamics and no noise

$\delta$	Double-blind	Open review			
	$$\mu $$				
	$\mu =0$	$\mu =0.2$	$\mu =0.4$	$\mu =0.6$	$\mu =0.8$
$\delta =0.1$	0.301	0.301	0.700	0.700	0.700
$\delta =0.2$	0.301	0.301	0.301	0.700	0.700
$\delta =0.3$	0.301	0.301	0.301	0.301	0.700

**Table 20:** Expected values of threshold levels (reviewers) computed for $\epsilon =0$ over simulations lasting 13,000 rounds with best response dynamics and no noise

$\delta$	Double-blind	Open review			
	$$\mu $$				
	$\mu =0$	$\mu =0.2$	$\mu =0.4$	$\mu =0.6$	$\mu =0.8$
$\delta =0.1$	0.200	0.200	0.700	0.700	0.700
$\delta =0.2$	0.200	0.200	0.200	0.699	0.700
$\delta =0.3$	0.200	0.200	0.200	0.200	0.699

**Table 21:** Expected values of effort levels (authors) computed for $\epsilon =0.1$ over simulations lasting 13,000 rounds with best response dynamics and no noise

$\delta$	Double-blind	Open review			
	$$\mu $$				
	$\mu =0$	$\mu =0.2$	$\mu =0.4$	$\mu =0.6$	$\mu =0.8$
$\delta =0.1$	0.749	0.750	0.752	0.750	0.752
$\delta =0.2$	0.748	0.749	0.748	0.752	0.749
$\delta =0.3$	0.750	0.751	0.746	0.751	0.748

**Table 22:** Expected values of threshold levels (reviewers) computed for $\epsilon =0.1$ over simulations lasting 13,000 rounds with best response dynamics and no noise

$\delta$	Double-blind	Open review			
	$$\mu $$				
	$\mu =0$	$\mu =0.2$	$\mu =0.4$	$\mu =0.6$	$\mu =0.8$
$\delta =0.1$	0.200	0.200	0.700	0.700	0.700
$\delta =0.2$	0.200	0.200	0.200	0.699	0.700
$\delta =0.3$	0.200	0.200	0.200	0.200	0.699

**Table 23:** Expected values of effort levels (authors) computed for $\epsilon =0.2$ over simulations lasting 13,000 rounds with best response dynamics and no noise

$\delta$	Double-blind	Open review			
	$$\mu $$				
	$\mu =0$	$\mu =0.2$	$\mu =0.4$	$\mu =0.6$	$\mu =0.8$
$\delta =0.1$	0.800	0.800	0.800	0.800	0.800
$\delta =0.2$	0.800	0.800	0.800	0.800	0.800
$\delta =0.3$	0.800	0.800	0.800	0.800	0.800

**Table 24:** Expected values of threshold levels (reviewers) computed for $\epsilon =0.2$ over simulations lasting 13,000 rounds with best response dynamics and no noise

$\delta$	Double-blind	Open review			
	$$\mu $$				
	$\mu =0$	$\mu =0.2$	$\mu =0.4$	$\mu =0.6$	$\mu =0.8$
$\delta =0.1$	0.200	0.200	0.700	0.700	0.700
$\delta =0.2$	0.200	0.200	0.200	0.699	0.700
$\delta =0.3$	0.200	0.200	0.200	0.200	0.699

**Table 25:** Expected values of effort levels (authors) computed for $\epsilon =0$ over simulations lasting 13,000 rounds with best response dynamics and noise

$\delta$	Double-blind	Open review			
	$$\mu $$				
	$\mu =0$	$\mu =0.2$	$\mu =0.4$	$\mu =0.6$	$\mu =0.8$
$\delta =0.1$	0.303	0.303	0.699	0.699	0.699
$\delta =0.2$	0.303	0.303	0.303	0.699	0.699
$\delta =0.3$	0.303	0.303	0.303	0.303	0.303

**Table 26:** Expected values of threshold levels (reviewers) computed for $\epsilon =0$ over simulations lasting 13,000 rounds with best response dynamics and noise

$\delta$	Double-blind	Open review			
	$$\mu $$				
	$\mu =0$	$\mu =0.2$	$\mu =0.4$	$\mu =0.6$	$\mu =0.8$
$\delta =0.1$	0.202	0.202	0.698	0.698	0.698
$\delta =0.2$	0.202	0.202	0.202	0.698	0.698
$\delta =0.3$	0.202	0.202	0.202	0.202	0.202

**Table 27:** Expected values of effort levels (authors) computed for $\epsilon =0.1$ over simulations lasting 13,000 rounds with best response dynamics and noise

	Double-blind	Open review			
	$$\mu $$				
$\delta$	$\mu =0$	$\mu =0.2$	$\mu =0.4$	$\mu =0.6$	$\mu =0.8$
$\delta =0.1$	0.748	0.750	0.748	0.749	0.747
$\delta =0.2$	0.749	0.749	0.749	0.749	0.749
$\delta =0.3$	0.749	0.747	0.747	0.749	0.749

**Table 28:** Expected values of threshold levels (reviewers) computed for $\epsilon =0.1$ over simulations lasting 13,000 rounds with best response dynamics and noise

$\delta$	Double-blind	Open review			
	$$\mu $$				
	$\mu =0$	$\mu =0.2$	$\mu =0.4$	$\mu =0.6$	$\mu =0.8$
$\delta =0.1$	0.202	0.202	0.698	0.698	0.698
$\delta =0.2$	0.202	0.202	0.202	0.697	0.698
$\delta =0.3$	0.202	0.202	0.202	0.202	0.697

**Table 29:** Expected values of effort levels (authors) computed for $\epsilon =0.2$ over simulations lasting 13,000 rounds with best response dynamics and noise

$\delta$	Double-blind	Open review			
	$$\mu $$				
	$\mu =0$	$\mu =0.2$	$\mu =0.4$	$\mu =0.6$	$\mu =0.8$
$\delta =0.1$	0.798	0.798	0.798	0.798	0.798
$\delta =0.2$	0.798	0.798	0.798	0.798	0.798
$\delta =0.3$	0.798	0.798	0.798	0.798	0.798

**Table 30:** Expected values of threshold levels (reviewers) computed for $\epsilon =0.2$ over simulations lasting 13,000 rounds with best response dynamics and noise

$\delta$	Double-blind	Open review			
	$$\mu $$				
	$\mu =0$	$\mu =0.2$	$\mu =0.4$	$\mu =0.6$	$\mu =0.8$
$\delta =0.1$	0.202	0.202	0.698	0.698	0.698
$\delta =0.2$	0.202	0.202	0.202	0.697	0.698
$\delta =0.3$	0.202	0.202	0.202	0.202	0.697

## Results

We shall begin by summarising the impact of the player variables in our model: $\epsilon$ (determining author effort bonuses from publishing higher effort research), $\delta$ (determining reviewer costs) and $\mu$ (determining reputation bonuses for both players from tough reviewing under open review). Each of the $\epsilon$ values that we considered has a profound effect on the author’s incentives. Consequently, our model replicates the plausibly strong connection between reputation boosts for author effort and authors’ decisions to expend high levels of effort on their paper. In particular, we can use $\epsilon$ to define three personal incentive regions for the author. When $\epsilon = 0$, the reputation bonus with every considered $\delta$ value turns the author into an effort minimizer, since the reputation bonus for high effort publications is too low to incentivize above-minimum effort. This does *not* mean that authors are expending low levels of effort on their papers, because all the papers that we model have at least enough effort to “pass the desk” at good journals. However, it does mean that authors are choosing the lowest possible effort levels given the constraints posed by the editorial desk. When $\epsilon =0.1$, the publication bonus is high enough to make the author indifferent between effort levels as long as the reviewer accepts the paper. In other words, the author gets the same payoff from each acceptance irrespective of the effort level. This level of $\epsilon$ neither incentivizes effort minimization, nor effort maximization. The games with this value of $\epsilon$ have many weak pure strategy Nash equilibria that are not evolutionarily stable. Finally, when $\epsilon = 0.2$, the author becomes an effort maximizer, regardless of reviewer behaviour.

The general tendencies of $\delta$ also matched commonsense expectations: increasing reviewer costs discourages reviewer effort. Every value of $\delta$ that we simulated had the effect of incentivizing the reviewer to minimize the effort cost by adopting the lowest possible threshold. However, higher $\delta$ values create a stronger incentive to adopt the minimum threshold, since they increase the payoff advantages associated with effort-minimizing strategy. Thus, higher $\delta$ values require higher $\mu$ values to counteract the reviewer’s incentive to minimize effort and thus incentivize the reviewer to adopt a threshold above the minimum.

In the subsections below, we shall explain our results under the wide range of variations that we studied. For each value of $\epsilon$, we shall summarise our results for each simulation dynamic that we used. For each simulation dynamic, we shall describe the effects of various values of $\delta$, $\mu$, and random noise. We assumed that $\epsilon \le 2$, because only then could relatively low author effort be rational. Therefore, we have subsections for $\epsilon$ values of 0, 0.1, and 0.2. Recall that we use these $\epsilon$ to determine the properties of $\epsilon$ value areas. Thus, for example, if $\epsilon$ were close to 0, then there would be similar results to $\epsilon =0.$

### Scenario (1) $\epsilon =0$

#### Best Response Dynamics

Under $\epsilon =0$, the reputation effects for authors come *solely* from having published at all in a good journal (and reviewer’s reports under open review) rather than assessments of their effort in papers. Put another way, in that case, journal reputations and reviewer reports are always used as proxies for assessing authors’ efforts. Under double-blind review ($\mu =0$) and with any considered $\delta$ value, the game has a unique strict pure strategy Nash equilibrium associated with the effort-threshold pair $\left( 0.3,0.2\right)$. For authors and reviewers, 0.3 and 0.2 are respectively their lowest possible values for their effort and threshold. We can interpret this result as showing that reviewers alone are not enough to raise author effort levels in our model: reputation boosts for effort are necessary. This Nash equilibrium is a regular and unique evolutionarily stable state under best response dynamics, with and without exogenous noise. In our simulations with best response dynamics, the population quickly converged to this state. The speed is reflected by the average effort and threshold levels provided in Tables 19 and 20.

How does open review impact the players’ behaviour under best response dynamics? The exact values of $\mu$ proved to make notable and complex differences to this impact. For each considered $\delta$ value, we can identify three subsets of considered $\mu$ values. For each $\delta$ value, there is a positive value $\mu ^{*}_{\delta }\in \left\{ 0.2,0.4,0.6,0.8\right\}$ that transforms the original game into a game with two pure strategy Nash equilibria. The first is a strict equilibrium associated with the effort-threshold pair $\left( 0.3,0.2\right)$. The second is a weak equilibrium associated with the pair $\left( 0.7,0.7\right)$, which occurs when $\mu ^{*}_{0.1}=0.2$, $\mu ^{*}_{0.2}=0.4$ or $\mu ^{*}_{0.3}=0.6$. This weak Nash equilibrium is not evolutionarily stable under best response dynamics. Hence, the population quickly converges to the only evolutionarily stable state, such that authors adopt the minimum effort level 0.3 and reviewers adopt the minimum threshold 0.2. For every $\delta$ value, every $\mu <\mu ^{*}_{\delta }$ generates a game which has the same unique strict Nash equilibrium as the corresponding double-blind game. Finally, each considered $\mu >\mu ^{*}_{\delta }$ generates a game with three or four^16^ strict Nash equilibria. Each of these games retains a strict Nash equilibrium associated with the effort-threshold pair $\left( 0.3,0.2\right)$. However, these games always have two additional strict Nash equilibria associated with the effort-threshold pairs $\left( 0.8,0.7\right)$ and $\left( 0.7,0.7\right)$. Both of them are strictly Pareto superior to the equilibrium associated with the pair $\left( 0.3,0.2\right)$ (i.e. they yield each player a strictly higher payoff), but one is not Pareto superior to another – the equilibrium associated with the pair $\left( 0.7,0.7\right)$ yields a strictly higher payoff to author than the equilibrium associated with the pair $\left( 0.8,0.7\right)$, while the equilibrium associated with the pair $\left( 0.8,0.7\right)$ yields a strictly higher payoff to the reviewer than the equilibrium associated with the pair $\left( 0.7,0.7\right).$

What about the stability of these Nash equilibria? The game has three or four evolutionarily stable states. Simulations show that, from our chosen initial population state where strategies are equally represented, the populations converge to an evolutionarily stable state associated with the effort-threshold pair $\left( 0.7,0.7\right)$. This is clearly visible in Tables 19, 20, 25 and 26: for values $\mu >\mu ^{*}_{\delta }$, we see a sharp increase in authors’ average effort level and reviewers’ average threshold.

Finally, we consider the effect of exogenous noise on our best response simulations. Exogenous noise affects neither the population dynamic, nor average author effort, nor reviewers’ threshold levels: compare Tables 19 to 24 with Tables 25 to 30. Therefore, noise was unimportant using best response dynamics.

#### Logit Response Dynamics

Although in general logit response dynamics resembles a smoothed version of best response dynamics in our simulations, there are some substantial differences. With all $\delta$ values under double-blind review ($\mu =0$), the population of reviewers converges to a state where the majority adopt the minimum threshold 0.2. When $\delta$ is 0.1, the population of authors converges to a state where an absolute majority of the population adopts effort level 0.6. Although the shares of players who adopt effort levels higher than 0.6 become negligible in the long run, substantial proportions of the population continue using effort levels 0.7 and 0.8 for considerable periods of time. Therefore, the average effort level under logit response dynamics is higher than 0.6. When $\delta$ is 0.2 and 0.3 under double-blind review, the population converges to a state where the majority of the population of authors adopts effort level 0.6, but a smaller yet non-insignificant proportion of the population adopts effort 0.5. The shares of populations using effort levels higher than 0.6 disappear quicker. Thus, the average effort level in the population drops as $\delta$ increases.

For all $\delta$ values, all values of $\mu \le \mu _{\delta }^{*}$ had no noticeable effect on the population of reviewers: the population quickly converged to a state where the majority of players adopted the lowest threshold 0.2. Yet higher values of $\mu \le \mu _{\delta }^{*}$ created a minor increase of the average effort level in the population of authors. This is explained by the fact that, in our simulations, higher $\mu$ values resulted in authors adopting effort levels higher than 0.6 for longer, because higher $\mu$ values increased the payoff gains associated with high effort levels, and consequently increased the probability of their selection under logit response dynamics. The greater persistence of these strategies resulted in slightly higher average effort levels. There was also a slight increase in reviewer threshold levels. Due to stochastic nature of the process, these differences might be due to random variation. A large scale study involving large numbers of simulations and reliable estimates of statistical errors is necessary to determine whether these results track a statistically significant tendency.

Finally, for each $\delta$ value, $\mu >\mu _{\delta }^{*}$ had a significant effect on the population of reviewers: the population quickly converged to a state where the absolute majority of players adopted threshold 0.7. The demanding population of reviewers quickly pushed the population of authors into a state where the absolute majority of players adopted a level of 0.7. This is noticeable in Tables 7 and 8 and 13 to 14: the smallest value of $\mu$ that exceeds the value of $\mu _{\delta }^{*}$ generates a sharp increase in the average effort. The average effort and threshold results of logit choice with and without noise were very similar, while the dynamics are virtually identical. Thus, the results seem robust with respect to low exogenous noise.

### Scenario (2) $\epsilon =0.1$

#### Best Response Dynamics

With all $\delta$ values, under double-blind review ($\mu =0$) the game has six weak Nash equilibria. In each equilibrium, the reviewer adopts the minimum threshold 0.2, while the author adopts one of the 6 effort levels. For the reviewer, a strategy adopting minimum threshold strictly dominates all others. Thus, the population of reviewers converges to a state where they adopt the minimum threshold of 0.2. The author receives the same payoff from each of the weak Nash equilibria in this game. However, the author’s equilibrium strategy determines the reviewer’s payoff. The pure strategy Nash equilibrium associated with the effort-threshold pairs $\left( 0.8,0.2\right)$ and $\left( 0.7,0.2\right)$ yield the reviewer strictly higher payoffs than the other pure strategy Nash equilibria. Although the Nash equilibrium associated with the effort-threshold pair $\left( 0.8,0.2\right)$ is weakly Pareto superior to equilibrium associated with the pair $\left( 0.7,0.2\right)$ – it yields the reviewer a strictly higher payoff – the author gets the same payoff in both. With our chosen tie-breaking rules and initial population state, the population of authors converges to a neutrally stable mixed population state^17^ in which half of the population adopts 0.7 and the other half adopts 0.8.^18^

The importance of $\mu$ for author and reviewer effort was varied. For all $\delta$ values, $\mu <\mu _{\delta }^{*}$ has no effect on the structure of the game or the dynamics. The game retains the same set of weak Nash equilibria. Under best response dynamics, the populations converge to the same states as under double-blind review. However, for every $\delta$ value, each $\mu _{\delta }^{*}$ transforms the original (i.e. double-blind review) game into a game with eight weak pure strategy Nash equilibria. In addition to the six Nash equilibria that are present under double-blind review, the game gets two additional weak pure strategy Nash equilibria associated with the effort-threshold pairs $\left( 0.8,0.7\right)$ and $\left( 0.7,0.6\right)$. Note that reviewer effort is higher in these Nash equilibria. However, because they are weak Nash equilibria, they do not survive. Interestingly, although the two new Nash equilibria yield the author a strictly higher payoff than the equilibria associated with the effort-threshold pairs $\left( 0.8,0.2\right)$ and $\left( 0.7,0.2\right)$, the two new equilibria yield the same payoff for the reviewer as equilibria associated with the pairs $\left( 0.8,0.2\right)$ and $\left( 0.7,0.2\right)$. Simulations proved to be very useful here, by revealing that the new equilibria have no effect on the ultimate results with best response dynamics: the populations converge to the same states as under double-blind review, and the average effort and threshold levels remain the same, see Tables 21 and 22.

Surprisingly, $\mu >\mu _{\delta }^{*}$ also had very limited effects on author effort levels. For all $\delta$ values, the values $\mu >\mu _{\delta }^{*}$ transform the original double-blind review game into a game with between six and seven Nash equilibria. Each of the new games has two strict Nash equilibria associated with the effort-threshold pairs $\left( 0.8,0.7\right)$ and $\left( 0.7,0.6\right)$. Both of these equilibria are strictly Pareto superior to others – they give both players strictly higher payoffs. Both of these equilibria yield the same payoff to the author, while for the reviewer, the Nash equilibrium associated with the pair $\left( 0.8,0.7\right)$ yields a strictly higher payoff than the one associated with the pair $\left( 0.7,0.6\right)$. Unsurprisingly, under best response dynamics, the population of reviewers converges to a state where they adopt a strategy associated with the maximum threshold 0.7. Meanwhile, the population of authors converges to a mixed population state where half of the population adopts effort level 0.8, while the other half adopts the level 0.7. As can be seen in Tables 21 to 22 and 27 to 28, a sufficiently high $\mu$ value generates a sharp increase in the average threshold in the population of reviewers. However, this has virtually no effect on the average effort level in the population of authors. Thus, with myopic payoff maximizing players, the effect of open review was undetectable under these conditions.

What was the effect of exogenous noise under these dynamics? We found that it has a negligible effect on the population dynamics in our model. This reflects the underlying robustness of the incentive structures described in this subsection. This stability is particularly interesting given our addition of exogenous noise.^19^ Under these dynamics and with this setting of $\epsilon =0.1$, the population converges to a neutrally stable state, rather than an evolutionarily stable state. In the latter condition, the population will revert to the state in response to an exogenous shock. By contrast, in a neutrally stable state, an exogenous shock can permanently shift the population away from the state; even if such shocks are unlikely, they might occur in the long run with random noise. However, in the particular case we studied, the low level of exogenous noise that we added to best response dynamics did not lead to a drift from the neutrally stable state. Hence, the robustness of our results to adding this noise level indicates their relative stability.

#### Logit Response Dynamics

Our simulations revealed substantial differences between best response and logit response dynamics. Under double-blind review, with all $\delta$ values, the population of reviewers quickly converged to a state where the absolute majority adopted the minimum threshold 0.2. The population of authors eventually converged to a state where the absolute majority of players adopted the second highest effort 0.7. However, the convergence was very slow. The population spent a substantial amount of time in a mixed population state where a substantial proportion of players adopted effort level 0.6. There was a lower average effort under double-blind review, as we can see in Tables 15 to 16 and 9 to 10, which was a result of that persistent mixed state.

What about open review under logit choice dynamics? For all $\delta$ values, the values $\mu \le \mu _{\delta }^{*}$ have no significant effect on the convergence of the population of reviewers: the population converges to a state where nearly everyone adopts the lowest threshold 0.2. Nonetheless, in our simulations, increasing $\mu$ values resulted in slightly higher average thresholds. This can be explained by the fact that, under logit choice dynamics, higher $\mu$ values allowed higher effort strategies to persist for longer, and thus convergence to the lowest effort state was slower. Notice that $\mu \le \mu _{\delta }^{*}$ had a more significant effect on the population of authors. This occurred because increasing $\mu$ values eliminated a mixed population state where a substantial proportion of players were using effort level 0.6, and shifted the population to a new mixed population state where the majority of players adopted effort 0.7, while a smaller yet quite significant proportion of players adopted effort 0.8. Unlike the proportion of players using effort 0.6, the proportion of players using effort level 0.8 did not vanish in the long run and increased with increasing $\mu$ values. These results are reflected in the average effort level which increased with increasing $\mu$ value: see Tables 15 to 16 and 9 to 10. However, the differences in values are relatively small. Furthermore, since the process is stochastic, only a large scale study can evaluate whether our simulations track a statistically significant tendency.

Finally, $\mu >\mu _{\delta }^{*}$ had a substantial effect on the population of reviewers. With these $\mu$ values, the population converged to a state where nearly everyone adopted the highest threshold. This result is reflected in average thresholds in the previously mentioned tables. With $\mu >\mu _{\delta }^{*}$, the population of authors was incentivized to converge to a population state where the majority of players adopted the highest effort level of 0.8. Due to the rapid convergence of reviewers to a high threshold state, the convergence in the population of authors was also rapid. As a consequence, there were considerably higher author average effort levels: see Tables 10 and 15. We observed similar patterns in logit response simulations with and without noise. Therefore, if $\epsilon$ is sufficiently large to counteract effort minimization, but not large enough to incentivize high effort, then open review can increase the effort levels of authors and increase reviewers’ expectations for high effort. Furthermore, even under assumptions of bounded rationality (modelled via logit choice) there is rapid convergence to this higher effort state.

### Scenario (3) $\epsilon =0.2$

#### Best Response Dynamics

Finally, we consider the case where $\epsilon =0.2$. Recall that, with greater values of $\epsilon$, authors invest a high level of effort regardless of what reviewers do. When $\epsilon =0.2$, the maximum effort level of 0.8 is an author’s strictly payoff dominant strategy under every combination of $\delta$ and $\mu$ values. Hence, in all cases under best response dynamics, the population of authors converges to a state where the population adopts the highest possible standard. The incentives of reviewers and, consequently, the structure of the game’s Nash equilibria depend on the values of $\delta$ and $\mu$. For each value $\delta$, the double-blind review game has a unique strict Nash equilibrium associated with the effort-threshold pair $\left( 0.8,0.2\right)$, since effort minimization is reviewer’s strictly payoff dominant strategy. Naturally, under best response dynamics, the population of reviewers converges to a state where the lowest threshold 0.2 takes over the population.

As one would expect, variations of $\mu$ affected reviewer behaviour, but not author behaviour. The structure of Nash equilibria remains the same for all values $\mu <\mu _{\delta }^{*}$. Each value $\mu _{\delta }^{*}$ transforms the original game into a game with two Nash equilibria – one strict Nash equilibrium associated with the effort-threshold pair $\left( 0.8,0.2\right)$ and a weak Nash equilibrium associated with the pair $\left( 0.8,0.7\right)$. The strict Nash equilibrium is the only evolutionarily stable state under best response dynamics, and so populations converge to the same states as under double-blind review: the population of authors adopts the highest effort level, 0.8, while the population of reviewers adopts the lowest threshold, 0.2. Meanwhile, with $\mu >\mu _{\delta }^{*}$, the original game is transformed into a game with a new unique and strict Nash equilibrium associated with the effort-threshold pair $\left( 0.8,0.7\right)$. Since this new equilibrium is the only evolutionarily stable state, the populations quickly converge to a state where authors adopt the highest effort while the reviewers adopt the highest threshold. The noise has a negligible effect on the population dynamic, see Tables 23 to 24 and 29 to 30.

#### Logit Response Dynamics

With $\epsilon =0.2$, logit response dynamics is a smooth close approximation of best response dynamics. With each combination of $\delta$ and $\mu$ value, the population of authors converges to a state where nearly everyone adopts the highest effort 0.8. The population of reviewers behaves in a similar manner as under best response dynamics: converges to the lowest threshold state under all values $\mu \le \mu ^{*}_{\delta }$ and to the highest threshold state when $\mu >\mu _{\delta }^{*}$. However, the values $\mu \le \mu _{\delta }^{*}$ had a small but noticeable effect on the population of reviewers: with higher $\mu$ values, the population’s convergence to the lowest threshold state was slower. Thus, higher threshold strategies survived in substantial numbers for longer periods of time. Therefore, while the impact of open review was not great with these values of $\mu$, it was identifiable. This is reflected by higher average thresholds with higher $\mu$ values: see Tables 17 to 18 and 11 to 12.

In addition, although $\epsilon =0.2$ creates a game where the author’s incentive to adopt the highest effort is independent from the reviewer’s actions, higher $\mu$ values had a small yet positive effect on the average effort in the population of reviewers. This can be explained by the fact that higher $\mu$ values enabled higher threshold levels in the population of reviewers to survive for longer in the initial stages of the evolutionary process, because reviewers under the logit choice protocol are more likely to pick such strategies when $\mu$ is greater. Thus, authors with low effort levels faced a higher rejection rate and were incentivised to adopt a higher effort level quicker than under lower levels of $\mu$.

Looking at our results in sum, we found that reviewer behaviour can make a difference to authors’ decisions about how much effort to invest in their papers, insofar as authors’ choices are not already determined by other factors in the model. Furthermore, we can see the effects of open review on effort within the period of our simulations. Open review provides reviewers with stronger incentives to require high levels of effort before they recommend acceptance. In disciplines and situations where journal reputations are used as reliable proxies for author effort, these reviewer incentives could be significant. However, even under double-blind review, lowering reviewer costs can have an effect in our model. In the real world, this could be via easier online systems, more accomodating review schedules, and so on. These can reduce reviewers’ incentives to minimise effort, and increase author effort in cases similar to $\epsilon =0.1$, i.e. when greater author effort in their published papers boosts their reputations, but not to the extent of dominating all other incentives.

## Discussion

Several general results from our simulations connect to contemporary debates about peer review:

1. Some have argued against peer review as an institution (Heesen & Bright, 2021). From the narrow perspective of encouraging author effort, peer review *does* make a positive difference in our model. More complex socio-political and methodological questions about peer review are beyond the scope of this article.
2. If we regard the quantity of time spent on peer review as a proxy for reviewer effort, then there is some empirical evidence that open review increases reviewer effort expenditure (van Rooyen et al., 2010). Our model provides a game theoretic mechanism for this effect, and using the same theoretical tools, it also provides a mechanism by which the increase in reviewer effort can lead to increased author effort.
3. The relative virtues of double-blind peer review versus open review are a longstanding topic of debate (see Sect.  1). Our model is naturally unable to capture all of the many dimensions of these controversies. However, with respect to incentivising author effort, we have found definite advantages to open review in our model under some conditions. In accordance with common sense intuitions, these advantages tend to be greater insofar as reviewer reputation bonuses *μ* are high relative to reviewer costs $\delta.$ They are especially important when authors’ reputation boosts for high effort publications are low. In the real world, there is little that can be done to reduce reviewer costs, but under open review, it is possible to directly link high effort reviewing to a reviewer’s reputation.

One might wonder whether there are ways to increase reviewer reputation bonuses within the double-blind review system. There are efforts to provide reputation bonuses for the aggregate *number* of contributions by a particular reviewer, such as the Publons scheme (Publons, 2022) but this only incentivises the number of reviews, and could even conceivably incentivise reviewers to produce large quantities of low-effort reviews, unless editors successfully control for quality. The same concern also arises for programmes such as *The Journal of Clinical Investigation*’s Review Rewards scheme, which offers guaranteed external review of one article for three reviews (Jackson, 2019). It also applies to the proposals of Kachewar and Sankaye (2013), who suggest a system for recognising reviewer contributions and tracking their reputations: incentivizing more reviewing, without reducing the costs of high reviewer thresholds, might undermine reviewers’ standards.

This concern is obviated by *The British Journal for the Philosophy of Science*’s “Referee of the Year” award (BSPS, 2019), which takes into account quality as well as quantity of reviewing.^20^ However, the reputation incentive from the Referee of the Year award seems weak: any single reviewer has a very low probability of receiving the award given the choice to produce a high effort review. Additionally, the award targets an overall year of review contributions rather than particular reviews, but reviewers do not know if they will be asked to review again that year, so there is a weak connection between (a) reviewers’ decisions for their commitment in a particular review and (b) their expectations of winning the award. Similar considerations apply to the *American Economic Review’s* “Excellence in Refereeing” award, which is given to “referees who have provided exceptional services to the Review by a large number and quality of referee reports” (American Economic Review, 2010).^21^

(4) Reputation bonuses for author effort, represented by $\epsilon$, can make a major difference in our model. In the age of “publish or perish”, increasing these bonuses could provide incentives for the exceptional effort that many reforms of science would require. There are variety of ways that these reputation bonuses could be increased in the real world. For instance, consider hiring, tenure, and funding decisions. If authorities making these decisions act in a way to reward high-effort research, even if it results in a comparatively terse publication record, then that could have a large impact. This would be analogous to raising $\epsilon$ in our model.

An example of an institutional move in this direction is a feature of the UK government’s Research Excellence Framework (previously “Research Assessment Exercise”) which is a regular governmental assessment of the UK’s departments with respect to their suitability for receiving future research funds (REF, 2021). Individual researchers are limited to submitting a maximum of five research items (books, articles etc.) for evaluation. Therefore, over the six year period since the last assessment in 2014, a researcher who published about one high effort research item per year would be more valuable to their department than a researcher who annually published even a large number of low quality research items. As a result, insofar as higher effort corresponds to assessments of research quality, higher effort researchers have (ceteris paribus) greater reputations within their departments and universities. In short, our model gives a reminder that peer review is not the only way to incentivise exceptional effort per-paper by scientists.

(5) Methodologically, we found that simulations were a suitable tool for evaluating the peer review systems’ effects on effort. Normally, in mechanism design, we can assume considerable control by the designer (or those whom they are advising) over the system being studied. However, in our study’s field of inquiry, journal editors, publishers, and other powerful figures within scientific publishing can choose among peer review systems, but not the evaluations of authors’ contributions by the scientific community. Thus, the reputation effects fall outside of designer’s control, and so optimal solutions of the model have no practical interest. This uncertainty also means that, instead of determining what will happen given a change of peer review system from double-blinding to open review, we must consider several plausible reactions by the community and simulate the consequences within our model.

(6) One concern that some scientists and editors have for open review is that early career researchers will be timorous with useful criticisms, especially when reviewing the research of more senior researchers. One possible system for dealing with this concern is “transparent peer review”, where reviewers are anonymous but the reports for accepted papers are published alongside the articles (Cosgrove & Flintoft, 2017; Pulverer, 2010). Assessing the overall value of different peer review systems is beyond our scope, but note that such a system would remove reviewers’ reputation bonuses under open review. In our model, this removal would entail the loss of robust incentives for author effort. As mentioned above, since reviews are only published for accepted articles in standard open review systems, there are small (at worst) disincentives for candid reviewing by early career researchers. It would be surprising if a significant number of authors would seek reprisals for critical but ultimately accepting reviews, so the dangers here seem to be low.

(7) With respect to the preceding points, it is important to recognise the limitations of our study. We have only considered one dimension of the peer review system: its role in encouraging author effort. Consequently, there are many aspects that our model does not capture, such as the fairness of the system. Some of these aspects might be tractable in future work. For instance, a very demanding peer review system might lead to reviewers and/or authors dropping out. Incorporating that possibility into our model would require very fundamental changes, including tracking players’ utilities and expanding their choice sets. Peer review is a complex social system that cannot be fully represented within any particular model, but we hope to be able to explore these further dimensions of peer review in future research.

An important proviso to all these points is that our model might not reflect the relevant realities of peer review. Nonetheless, it is helpful as a means of generating hypotheses for empirical investigation, identifying strategic possibilities, and enabling greater clarity in the formulation of different claims about the interactive effects of choices by authors and reviewers. Consider $\epsilon _i$, which is the multiplier of the chosen effort level of an author *i* that determines their reputation bonus from investing that effort into a published article. In the real world, the $\epsilon$ multiplier is determined by a variety of interacting factors. By taking $\epsilon$ as an exogenous variable, we have been able to develop a tractable exploratory model. In future research, it might be possible to make $\epsilon$ endogenous using insights from articles such as Chavalarias (2016), Parchomovsky (2000), Smaldino and McElreath (2016), and Tiokhin et al. (2021).

## Conclusions

We have demonstrated how game theory can help us to think about peer review institutions and how they affect research effort. Although we have utilised simulation methods, we have not sought to advance simulation methods at a technical level. Yet this familiarity is a strength, because our topic is so new that it would be risky to combine both novel methods and a novel domain. Our use of well-grounded tools removes this risk. Simulations proved to be useful methods for our study, due to multiple Nash equilibria, stochastic elements, and their graphical displays of the model’s evolutionary dynamics.

Considering our model as a whole, there is always a trade-off between tractability and detail. If we added many more variables, it would become extremely difficult to isolate the effects of changing any specific part of our model. However, to model double-blind and open review, we had to include enough variables to demarcate the primary effort differences in these systems’ incentive structures. Another aspect is relevance. Each variable proved to be important under some circumstances. For the variables $\delta$, $\mu$, and $\epsilon$, these circumstances were detailed in Sect.  4. For our randomness parameters, we know mathematically that setting these high makes player behaviour random, while setting them very low or to zero makes them approximate or act as best-response players. Therefore, there does not seem to be a simpler model with the same strengths.

There are several exciting areas in which our research can be extended. Firstly, editors are absent in our model, except insofar as we assume that they have already filtered out low-effort papers. Since their decisions affect those of authors and reviewers, it would be interesting to incorporate them into the model.^22^ Secondly, open review might have many reputation effects on reviewers, such as whether they acquire a reputation as a “tough” or “soft” reviewer, and our model could also be extended to incorporate these reputation effects. Thirdly, the separation of reviewers and authors into separate populations is a simplification that could be removed. Fourthly, peer review systems vary among scientific disciplines. For example, in physics and mathematics, postpublication reputation plays a major role in determining the professional benefits (or costs) of a paper; in contrast, in sciences like economics, the reputation of the journal is crucial for many funding and promotion decisions. The issue of prepublication versus postpublication review as levers for increasing author effort is a lively area of debate (Abdin et al., 2021; Heesen & Bright, 2021; Rowbottom, 2021).^23^ We look forward to investigating some of these issues in the future.

## Data Availability

Authors are available to provide employed data for this manuscript at their request.

## Footnotes

1 Note that replication problems vary among disciplines: experimental economics generally does better than many other areas of social science (Camerer et al., 2016) though improvement is nonetheless highly desirable.

2 A journal might also blind with respect to results, in order to reduce the bias in favour of statistically significant results (Glymour and Kawachi, 2005; Newcombe, 1987).

3 See Snodgrass (2006) for an extensive survey of relevant debates.

4 We do not assume the converse, that average or high effort papers are never desk rejected.

5 In a quasistrict Nash equilibrium, if there is a nonzero probability that any player will adopt an action, then that action must be strictly payoff dominant over any action that the player has zero probability of choosing.

6 For discussion and proofs, see Sandholm (2010a), Selten (1980) and Weibull (1995).

7 A potential game is one in which players’ incentives for strategy alterations can be expressed via a single function, called the potential function (Monderer and Shapley, 1996).

8 For discussion of the analytical techniques for potential and near-potential games, see Alós-Ferrer and Netzer (2010); Candogan et al. (2013); Lahkar and Riedel (2015).

9 For extensive discussions of the logit choice rule and logit response dynamics, see Alós-Ferrer and Netzer (2010), Blume (1997, 2003), Fudenberg and Levine (1998), Hofbauer and Sandholm (2002, 2007) and Mattsson and Weibull (2002).

10 A normal form game is one where we completely specify the possible strategies and payoffs for a finite number of players.

11 A richer model of scientists’ behaviour could disaggregate these costs, but we saw no advantage of including these details in this particular model, because the costs are mostly not determined by the structure of peer review systems.

12 One might argue that open reviewing exposes reviewers to retaliation costs from authors. For example, a senior academic may be able to punish a very junior academic in response to a negative review. However, under existing open review procedures, only the reviews of accepted articles are published. While authors might be frustrated by reviewer choices, such as repeated revise-and-resubmit requests, even for ultimately accepted articles, our topic is the effects of reviewers’ final decisions to accept or reject articles. We look forward to modelling revise-and-resubmits in future research.

13 We used submissions to economics journals as an inspiration for this figure. While data for the total number of submissions across all economics journals is unavailable, the top five journals received 1160 submissions each in 2011 (Card & DellaVigna, 2013). The number of submissions for low quality journals would naturally be far less, but in this article we are interested only in relatively high quality research.

14 For discussions of the properties of best response and logit dynamics, see Alós-Ferrer and Netzer (2010), Lahkar and Riedel (2015) and Sandholm (2015).

15 An alternative way to implement slightly imperfect rationality players would be to use replicator dynamics. However, replicator dynamics would translate each player’s payoff into the number of offspring that use the same strategy, so replicator dynamics would be contrary to our conception of players as imperfectly pursuing rationality. Nonetheless, our choice of a low logit noise level means that our model approximates replicator dynamics.

16 When $\delta =0.1$ and $\mu \in \left\{ 0.6,0.8\right\}$, the game has an additional fourth strict pure strategy Nash equilibrium associated with the effort-threshold pair $\left( 0.6,0.7\right)$. It is always strictly Pareto superior to the Nash equilibrium associated with the effort-threshold pair $\left( 0.3,0.2\right)$, but always strictly inferior to the Nash equilibria associated with the pairs $\left( 0.7,0.7\right)$ and $\left( 0.8,0.7\right)$.

17 That is, the population as a whole has multiple pure strategies and the population will not drift away from a stable state to any other Nash equilibrium.

18 In this mixed population state, a reviewer has a 1/2 probability of being matched with an author who adopts effort 0.7, as well as 1/2 probability of being matched with an author who adopts effort 0.8. As a result, each reviewer’s expected payoff from every random matching is the same.

19 We thank an anonymous reviewer for suggesting this point.

20 See also Siemens (2013).

21 There are also some reviewer incentive systems that require future modelling before they can be assessed (Johnston, 2015).

22 An anonymous referee helpfully points out that the editor can be understood as facing a monitoring problem regarding authors, with reviewers serving as intermediaries who themselves require monitoring. We look forward to expanding our model using this approach in future research.

23 We are grateful to an anonymous referee for this suggestion.

## Code Availability

Authors are available to provide employed code for this manuscript at their request.
